# Supplementary figures and images for: Model for finding the number of honey bee colonies needed for the optimal foraging process in a specific geographical location
Source: PeerJ. 2021 Sep 17;9:e12178. doi: 10.7717/peerj.12178 (PMC8451444; doi:10.7717/peerj.12178)

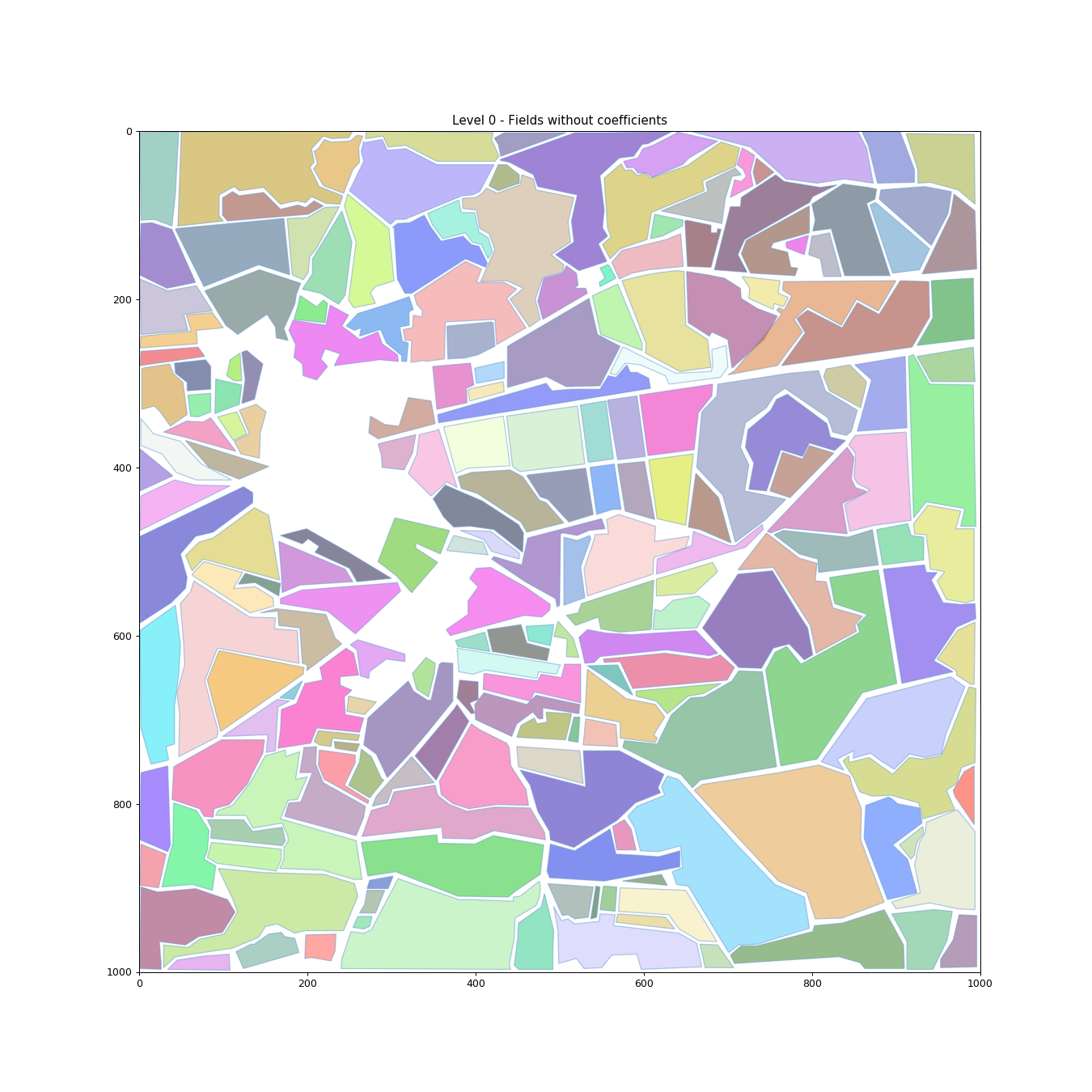

Supplement: Supplemental Information 1 — Python scripts developed to describe and run the model [file peerj-09-12178-s001.zip › Optimal Hives Amount in Point - Model/ClassLevelImages/Level 0 - Fields without coefficients.png]

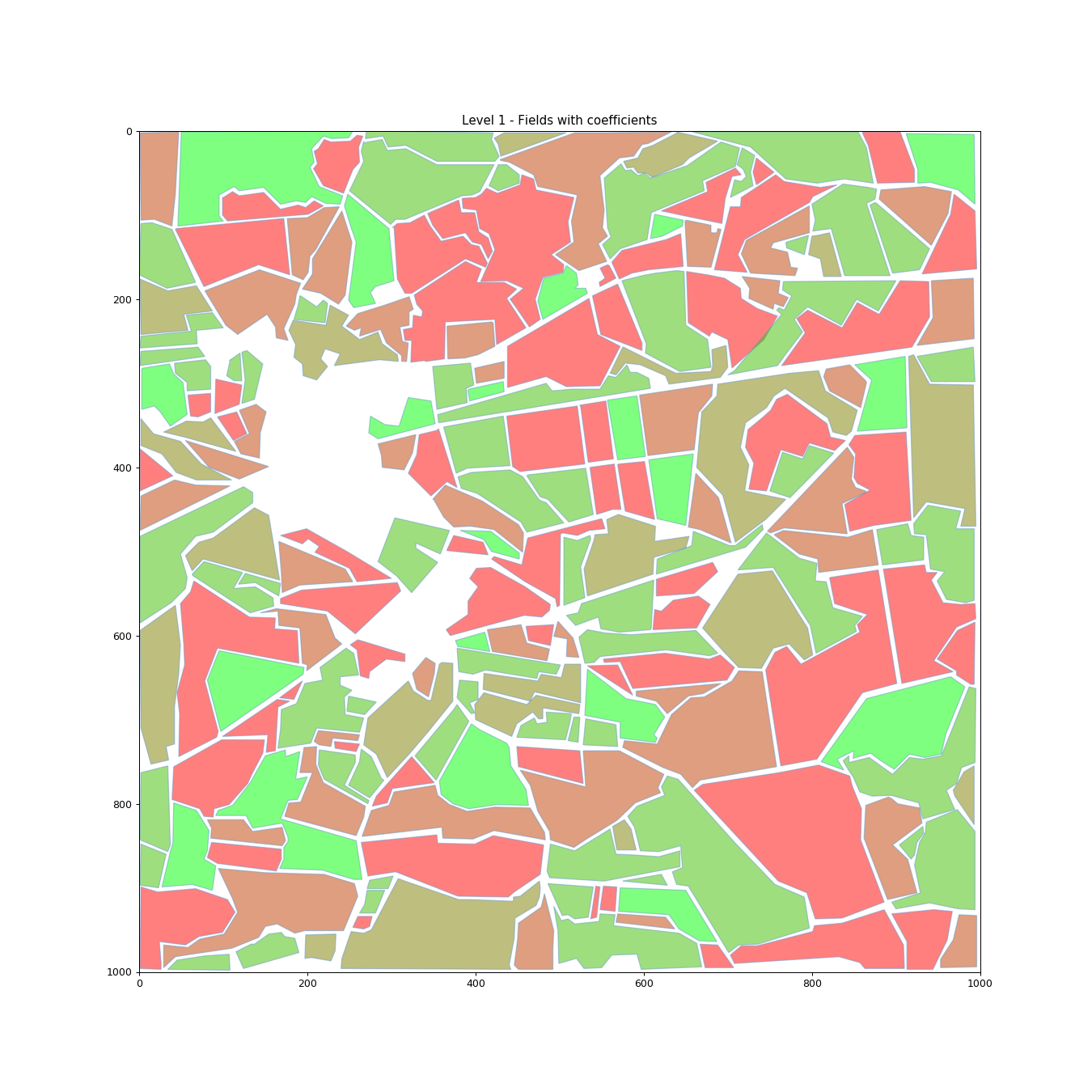

Supplement: Supplemental Information 1 — Python scripts developed to describe and run the model [file peerj-09-12178-s001.zip › Optimal Hives Amount in Point - Model/ClassLevelImages/Level 1 - Fields with coefficients.png]

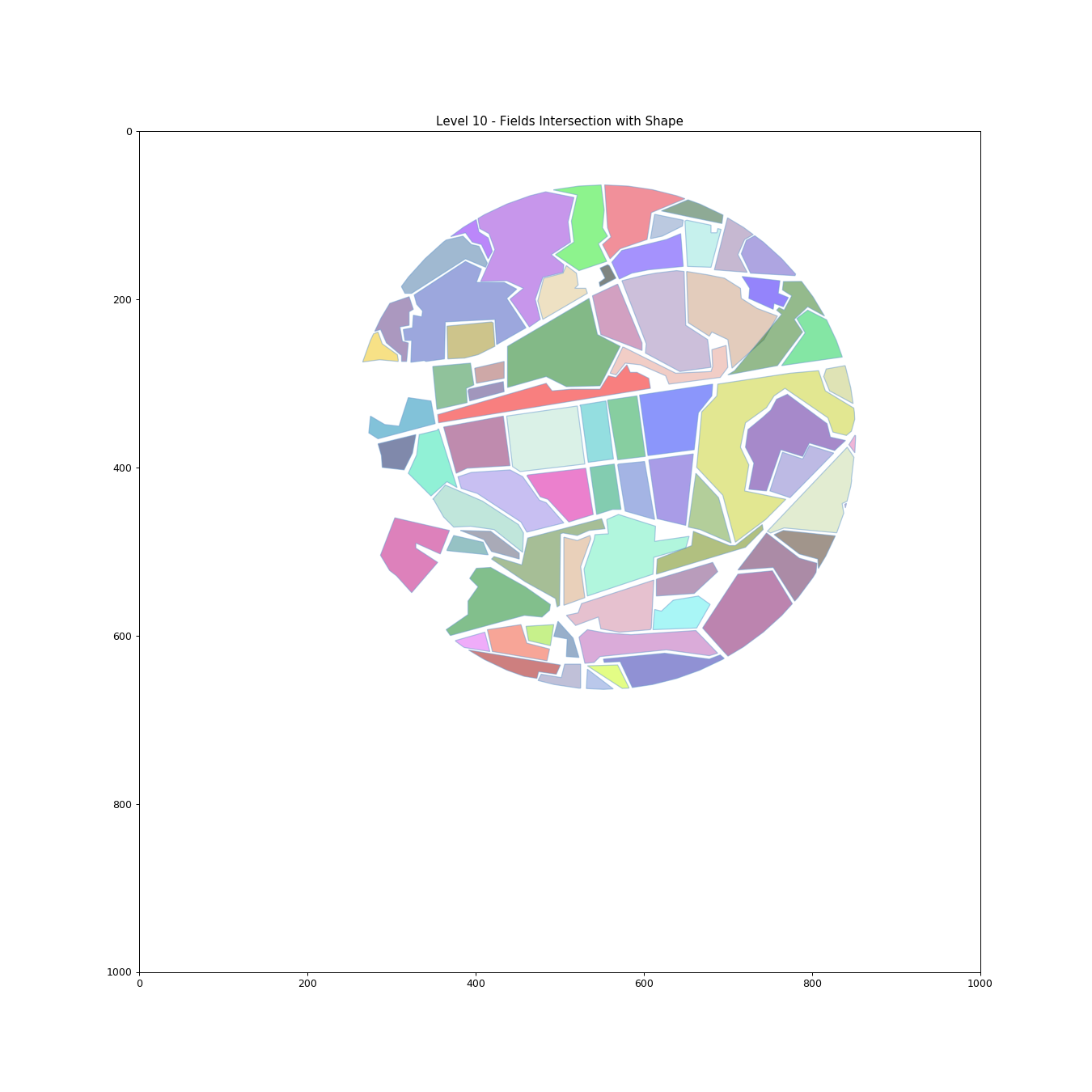

Supplement: Supplemental Information 1 — Python scripts developed to describe and run the model [file peerj-09-12178-s001.zip › Optimal Hives Amount in Point - Model/ClassLevelImages/Level 10 - Fields Intersection with Shape.png]

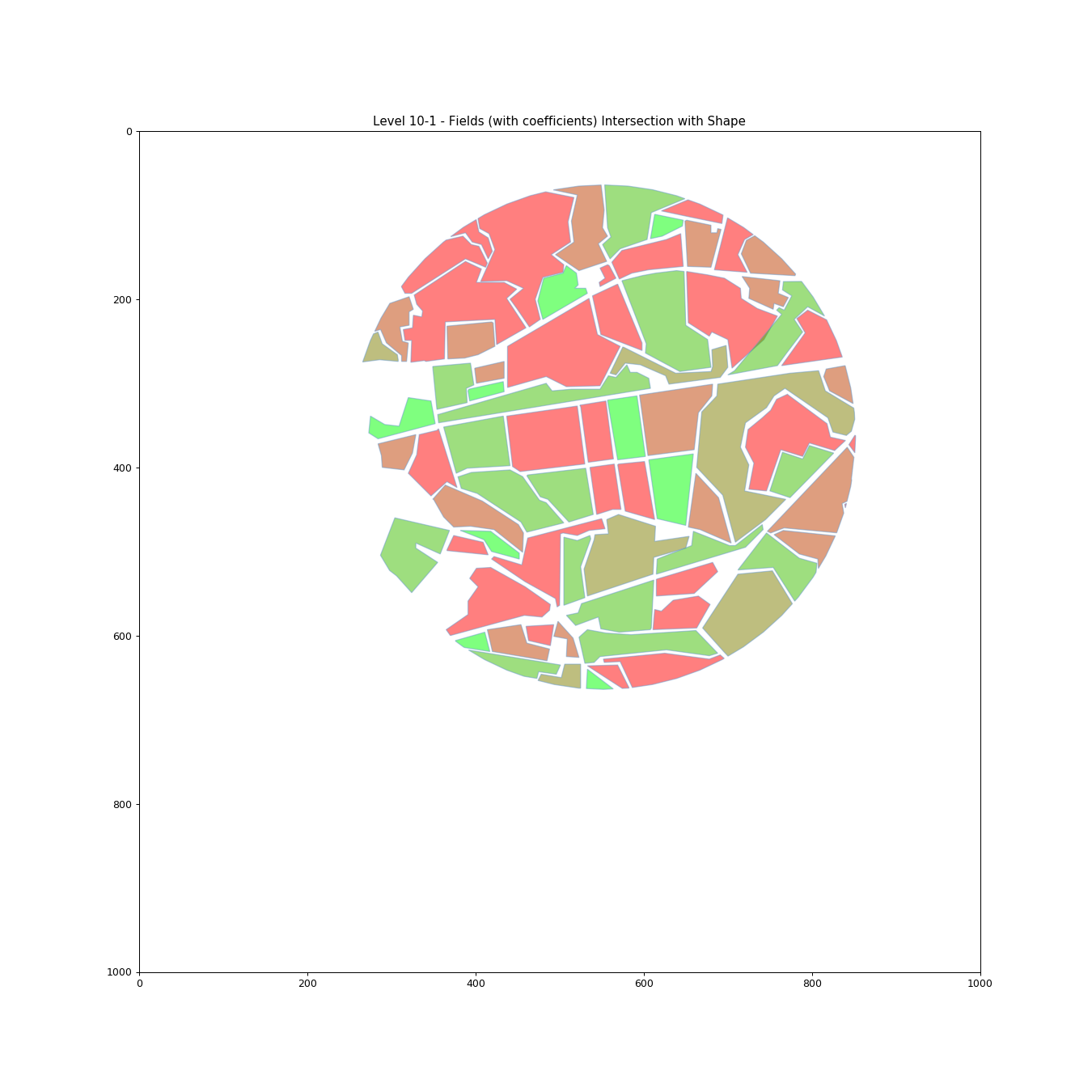

Supplement: Supplemental Information 1 — Python scripts developed to describe and run the model [file peerj-09-12178-s001.zip › Optimal Hives Amount in Point - Model/ClassLevelImages/Level 10-1 - Fields (with coefficients) Intersection with Shape.png]

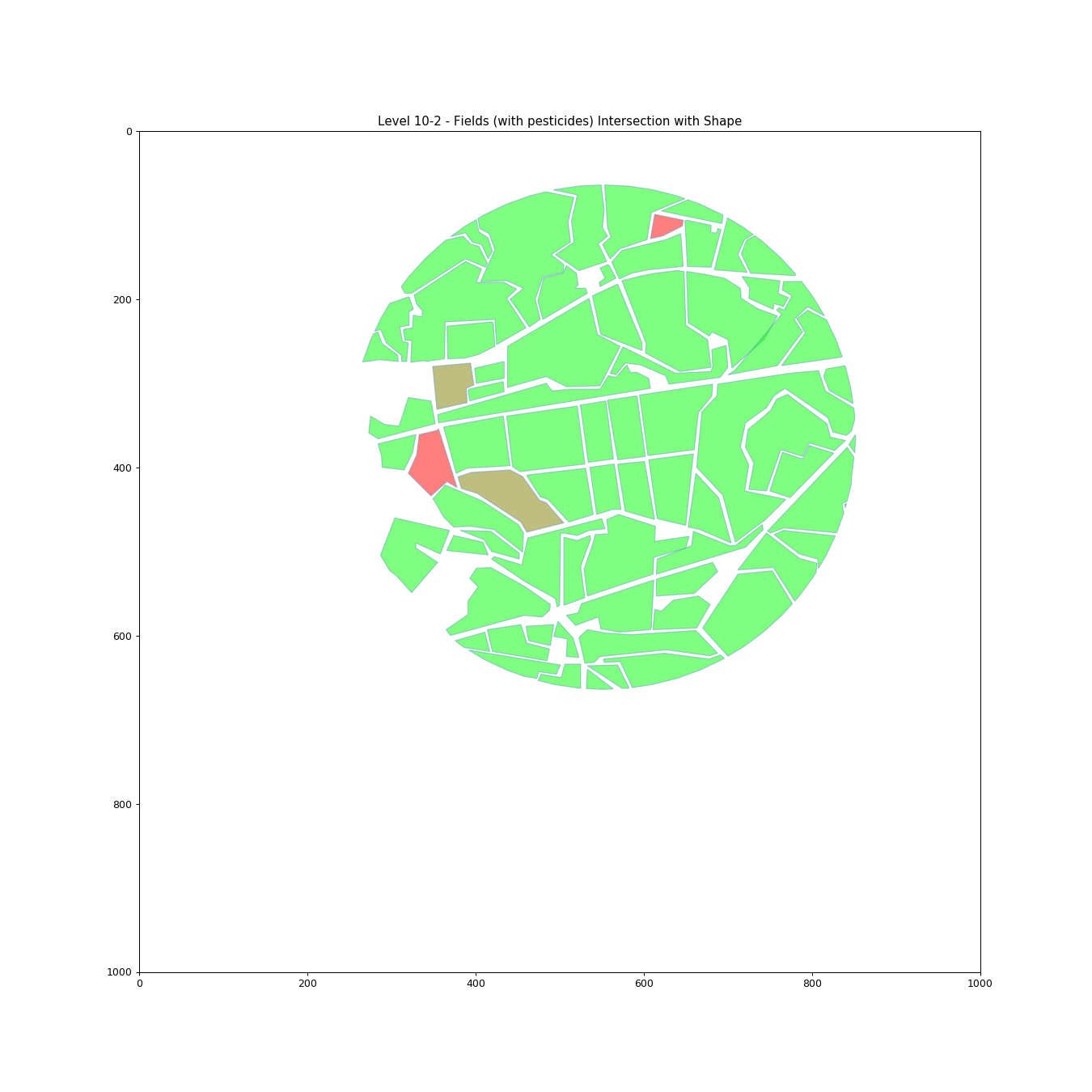

Supplement: Supplemental Information 1 — Python scripts developed to describe and run the model [file peerj-09-12178-s001.zip › Optimal Hives Amount in Point - Model/ClassLevelImages/Level 10-2 - Fields (with pesticides) Intersection with Shape.png]

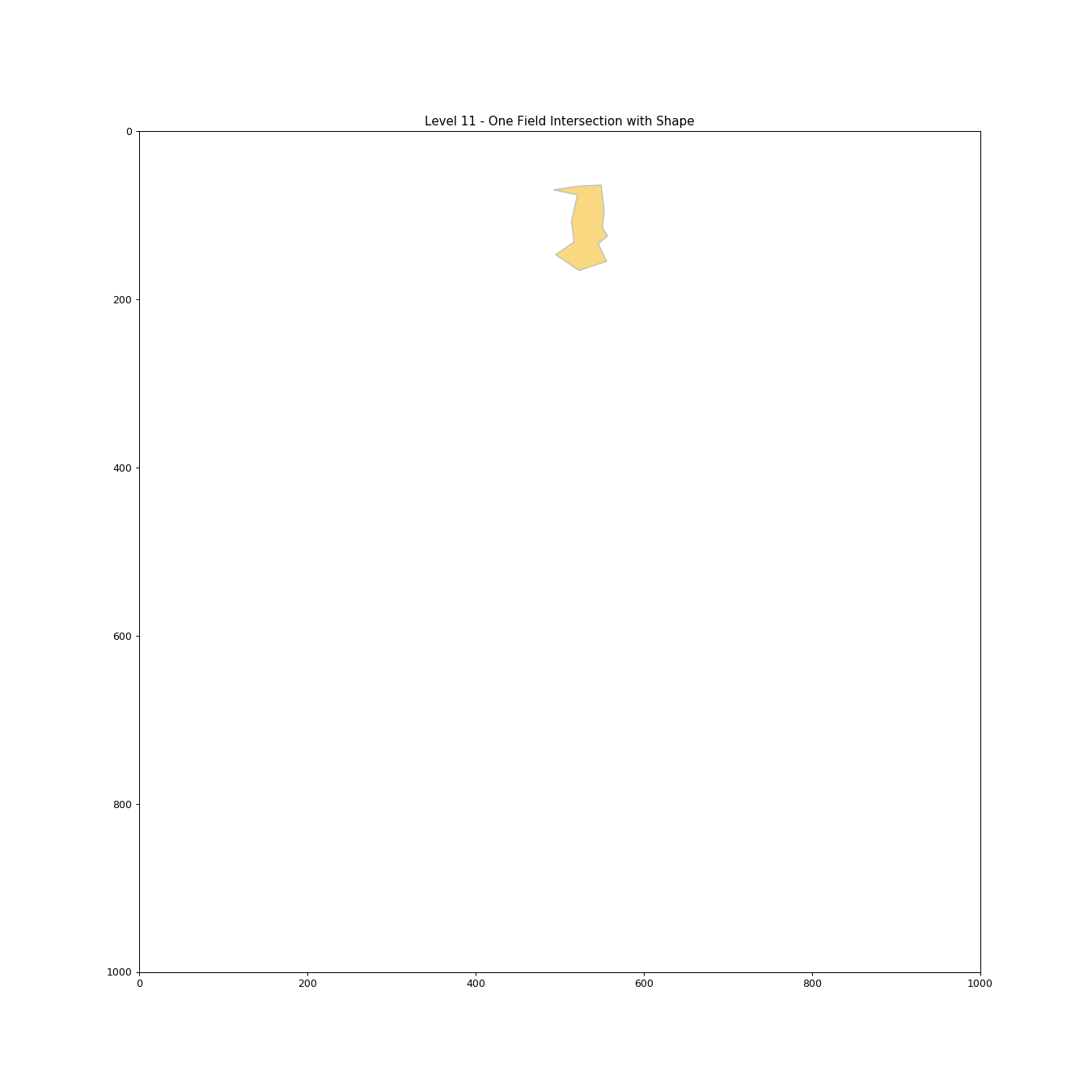

Supplement: Supplemental Information 1 — Python scripts developed to describe and run the model [file peerj-09-12178-s001.zip › Optimal Hives Amount in Point - Model/ClassLevelImages/Level 11 - One Field Intersection with Shape.png]

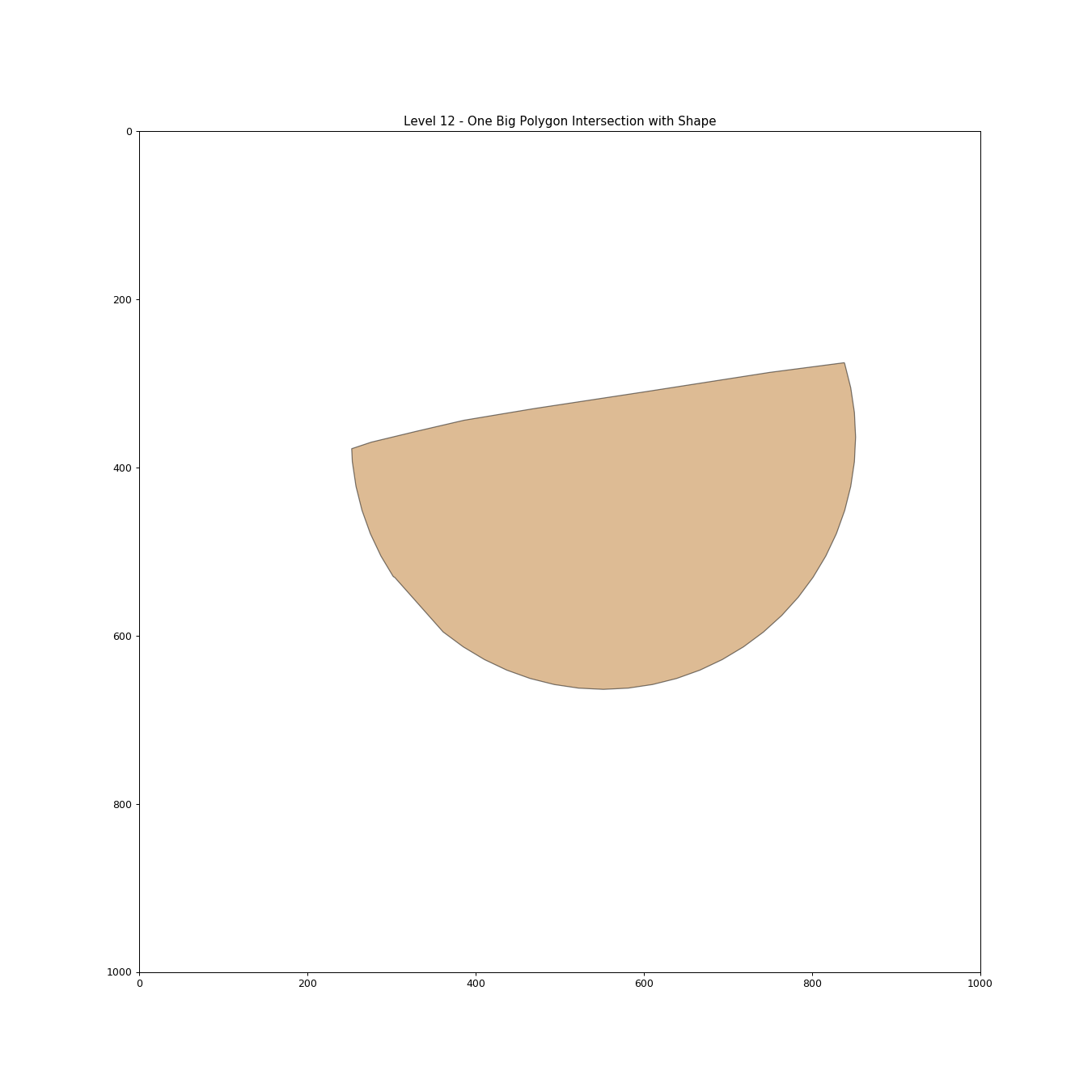

Supplement: Supplemental Information 1 — Python scripts developed to describe and run the model [file peerj-09-12178-s001.zip › Optimal Hives Amount in Point - Model/ClassLevelImages/Level 12 - One Big Polygon Intersection with Shape.png]

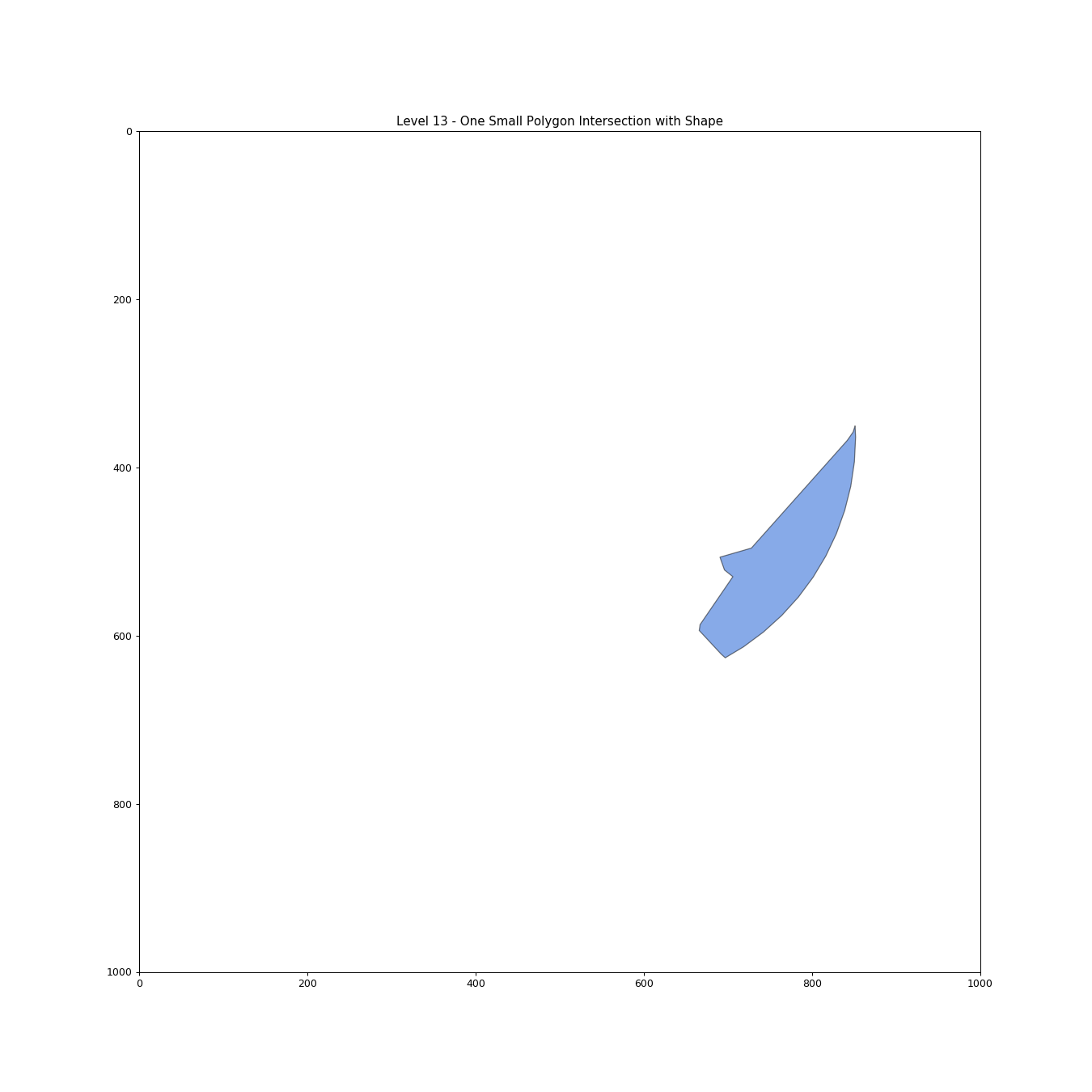

Supplement: Supplemental Information 1 — Python scripts developed to describe and run the model [file peerj-09-12178-s001.zip › Optimal Hives Amount in Point - Model/ClassLevelImages/Level 13 - One Small Polygon Intersection with Shape.png]

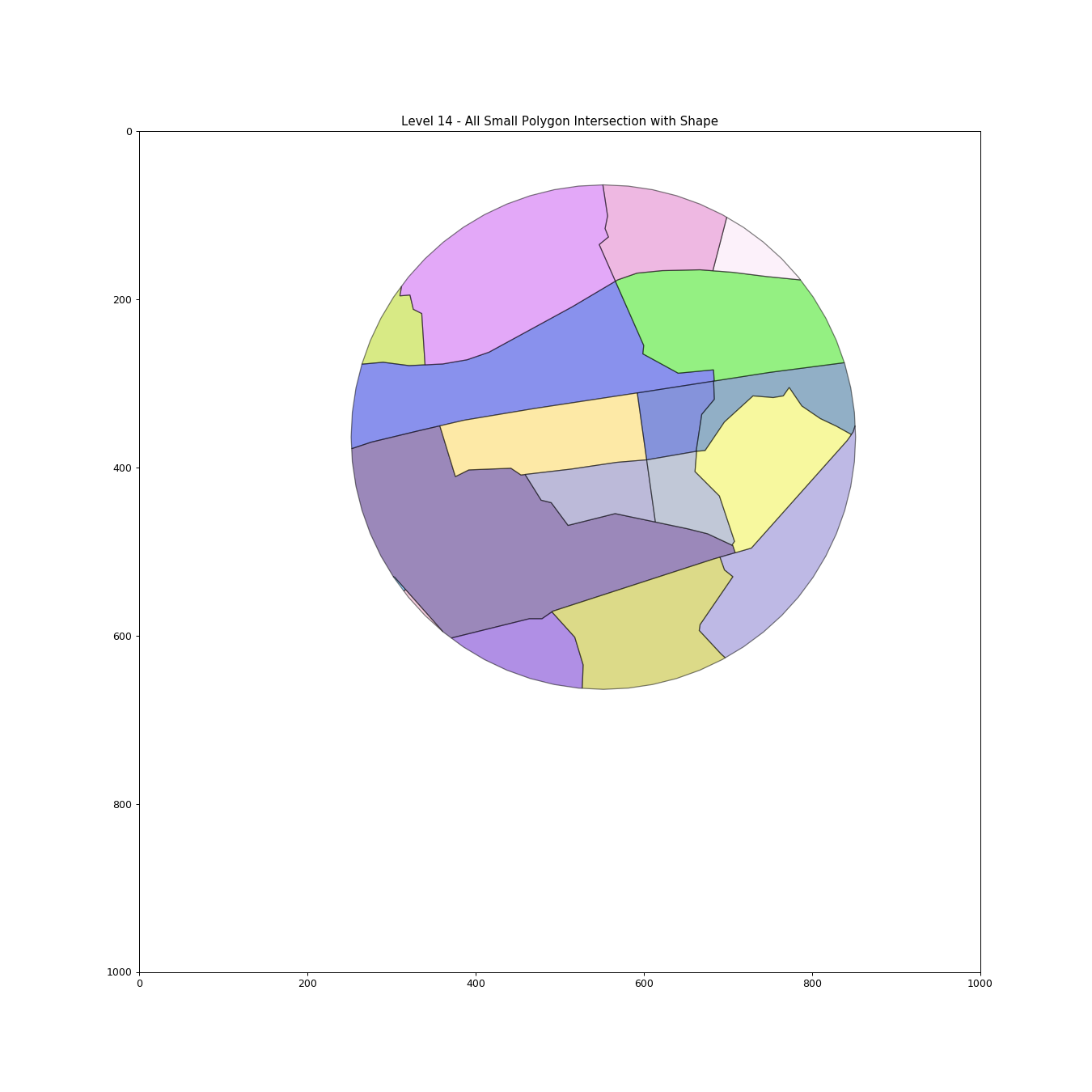

Supplement: Supplemental Information 1 — Python scripts developed to describe and run the model [file peerj-09-12178-s001.zip › Optimal Hives Amount in Point - Model/ClassLevelImages/Level 14 - All Small Polygon Intersection with Shape.png]

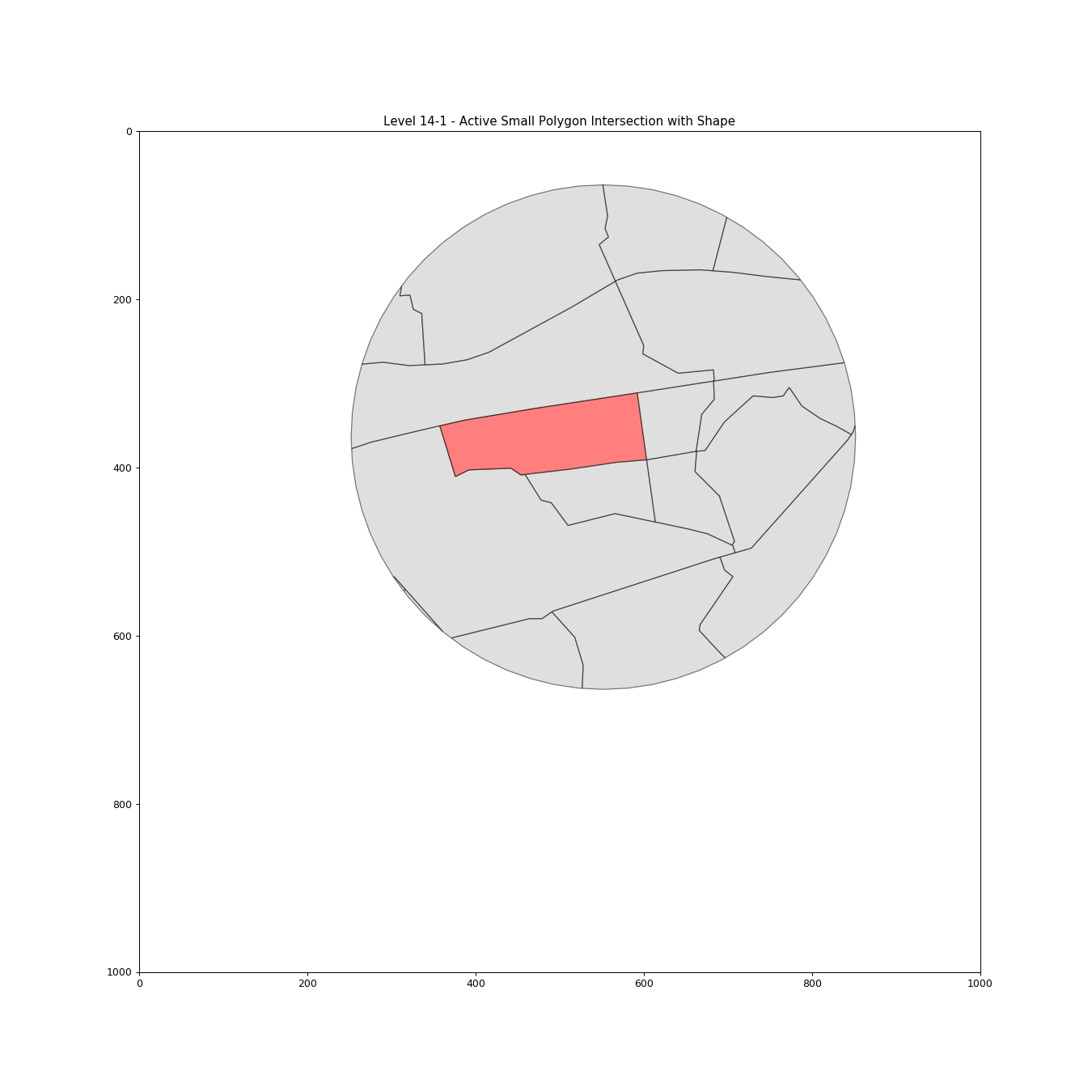

Supplement: Supplemental Information 1 — Python scripts developed to describe and run the model [file peerj-09-12178-s001.zip › Optimal Hives Amount in Point - Model/ClassLevelImages/Level 14-1 - Active Small Polygon Intersection with Shape.png]

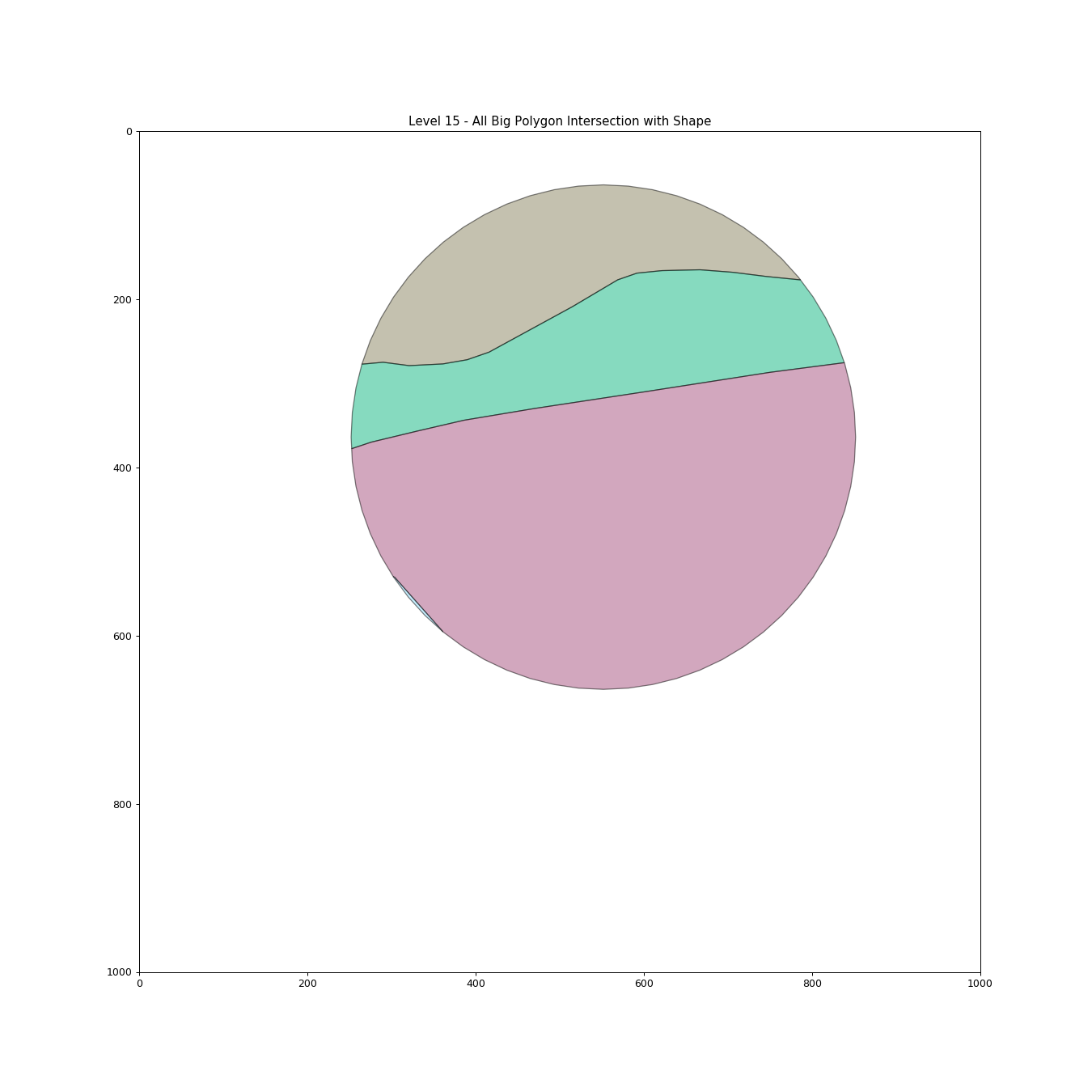

Supplement: Supplemental Information 1 — Python scripts developed to describe and run the model [file peerj-09-12178-s001.zip › Optimal Hives Amount in Point - Model/ClassLevelImages/Level 15 - All Big Polygon Intersection with Shape.png]

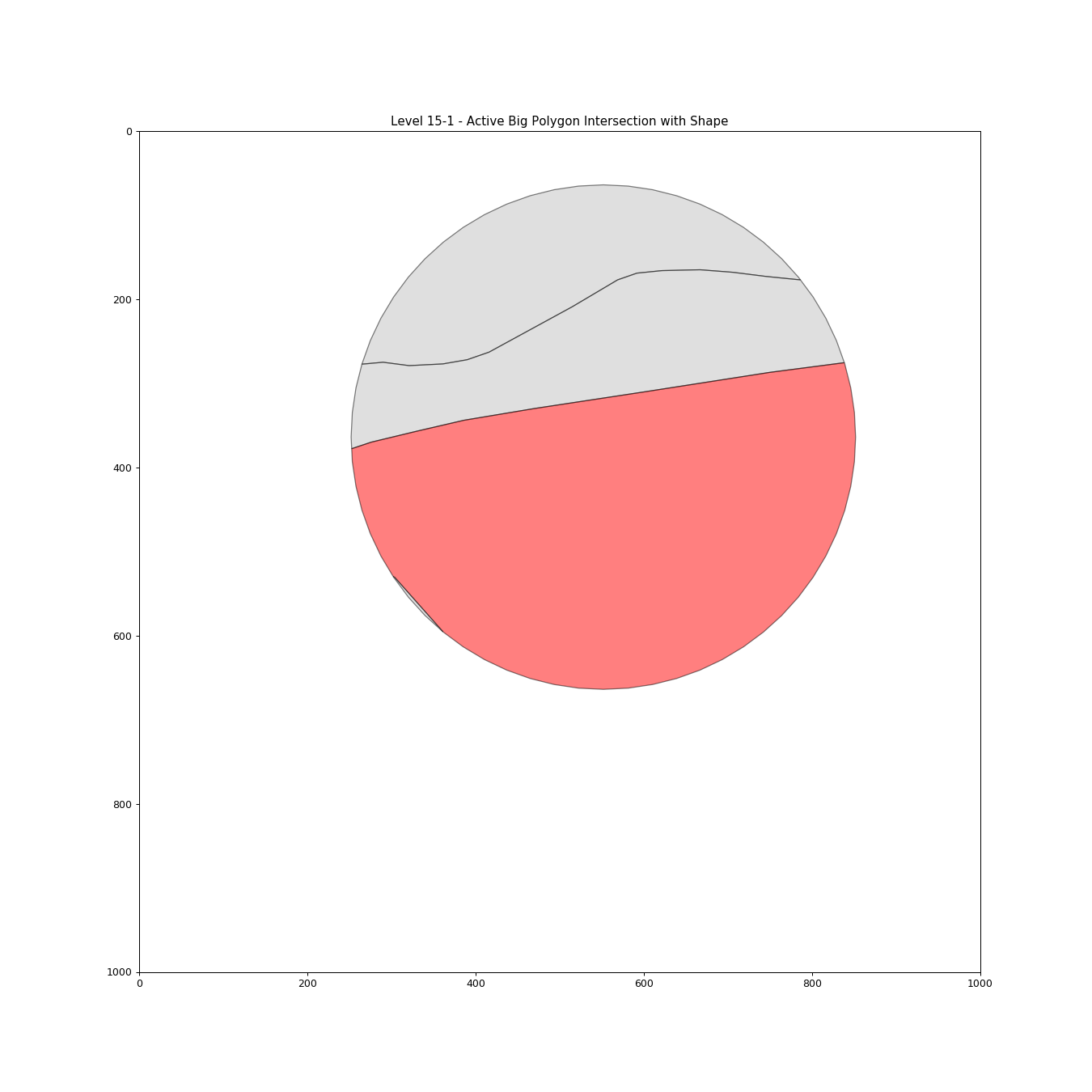

Supplement: Supplemental Information 1 — Python scripts developed to describe and run the model [file peerj-09-12178-s001.zip › Optimal Hives Amount in Point - Model/ClassLevelImages/Level 15-1 - Active Big Polygon Intersection with Shape.png]

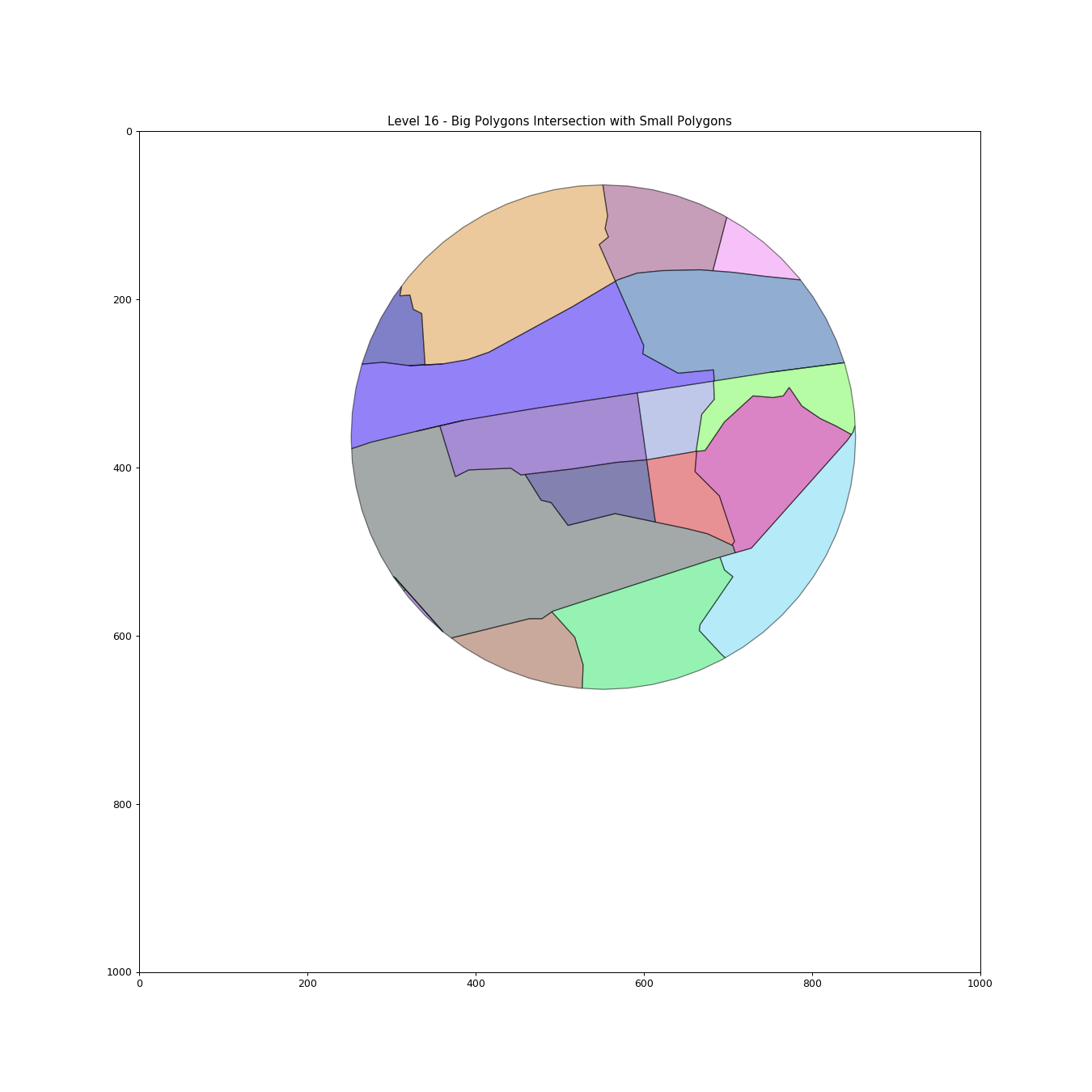

Supplement: Supplemental Information 1 — Python scripts developed to describe and run the model [file peerj-09-12178-s001.zip › Optimal Hives Amount in Point - Model/ClassLevelImages/Level 16 - Big Polygons Intersection with Small Polygons.png]

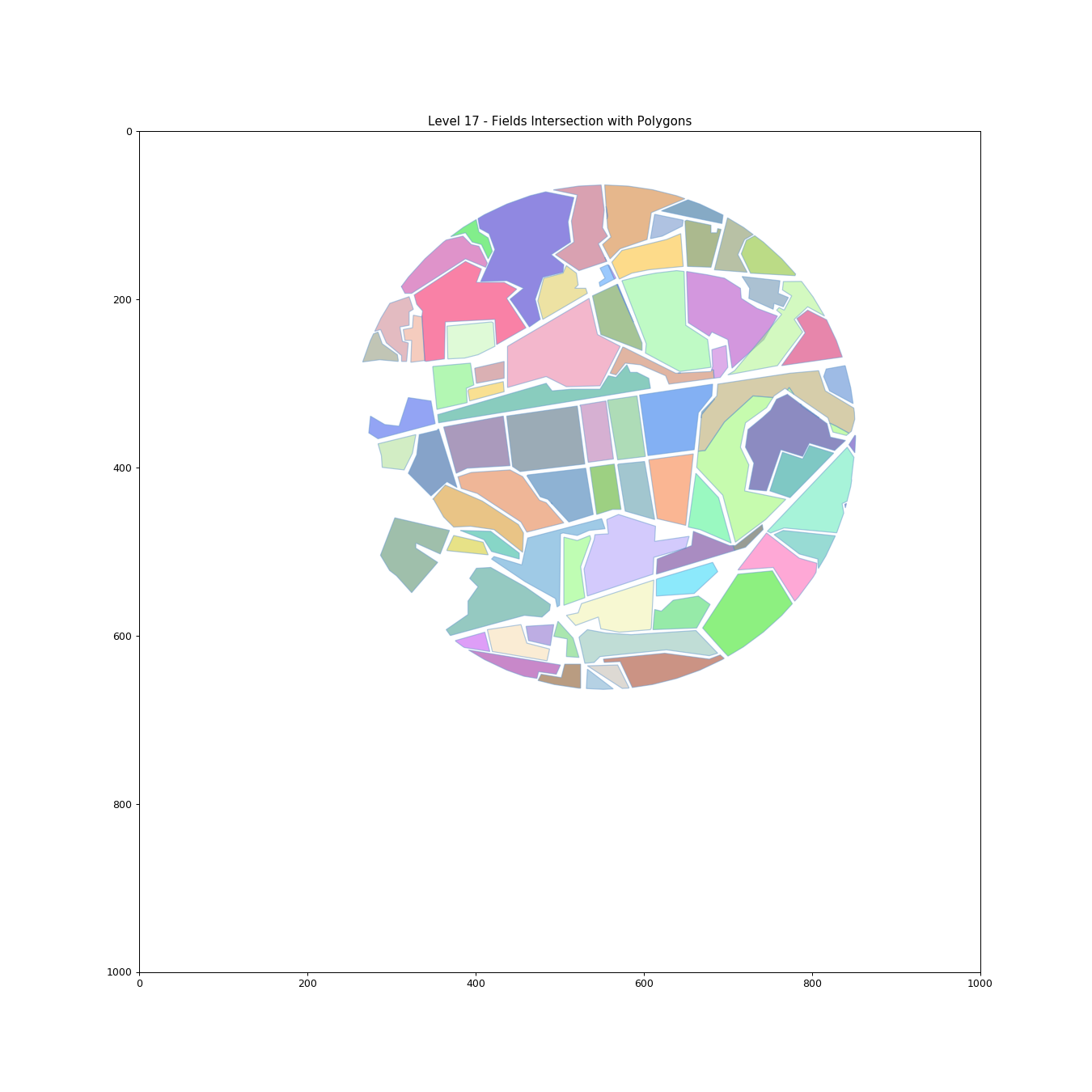

Supplement: Supplemental Information 1 — Python scripts developed to describe and run the model [file peerj-09-12178-s001.zip › Optimal Hives Amount in Point - Model/ClassLevelImages/Level 17 - Fields Intersection with Polygons.png]

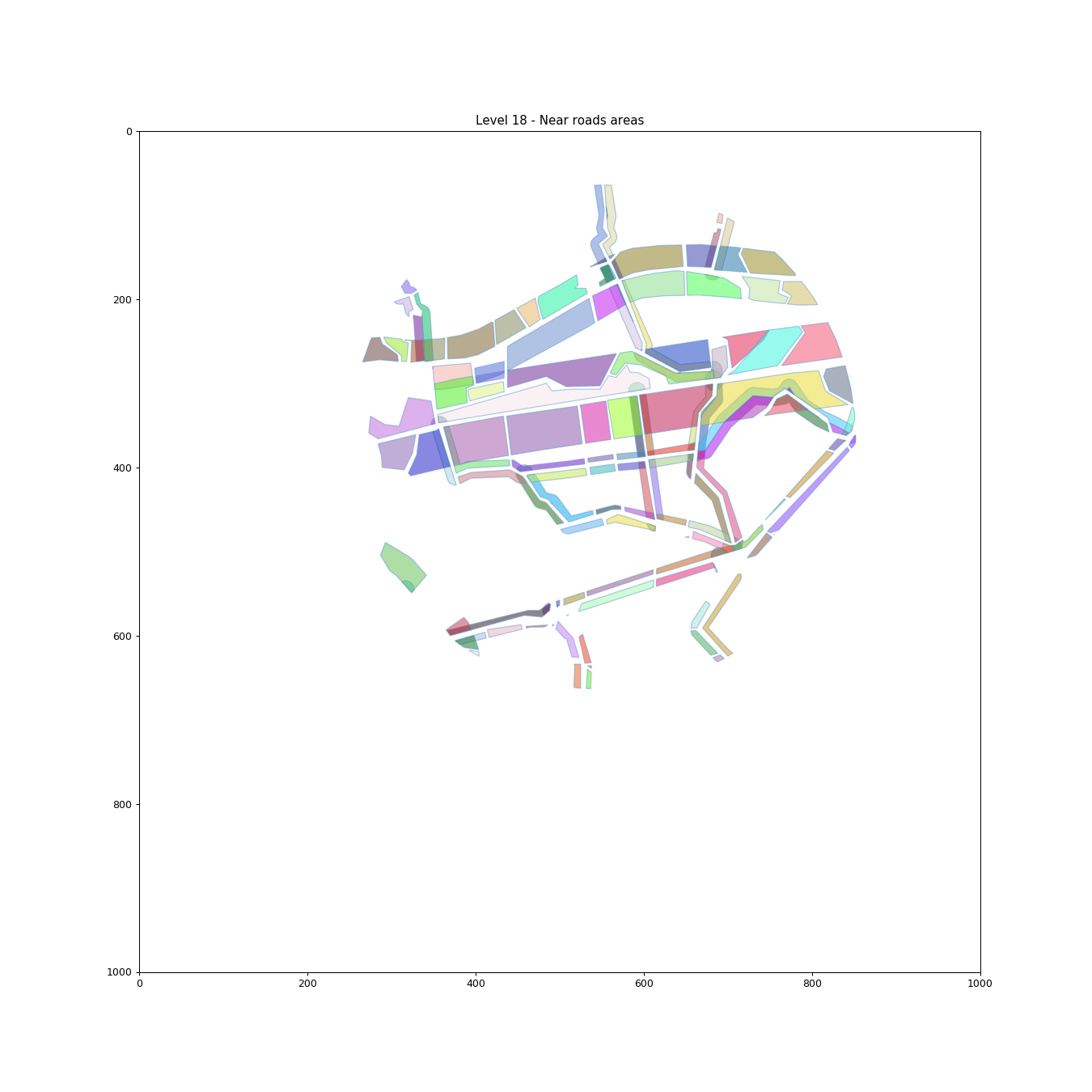

Supplement: Supplemental Information 1 — Python scripts developed to describe and run the model [file peerj-09-12178-s001.zip › Optimal Hives Amount in Point - Model/ClassLevelImages/Level 18 - Near roads areas.png]

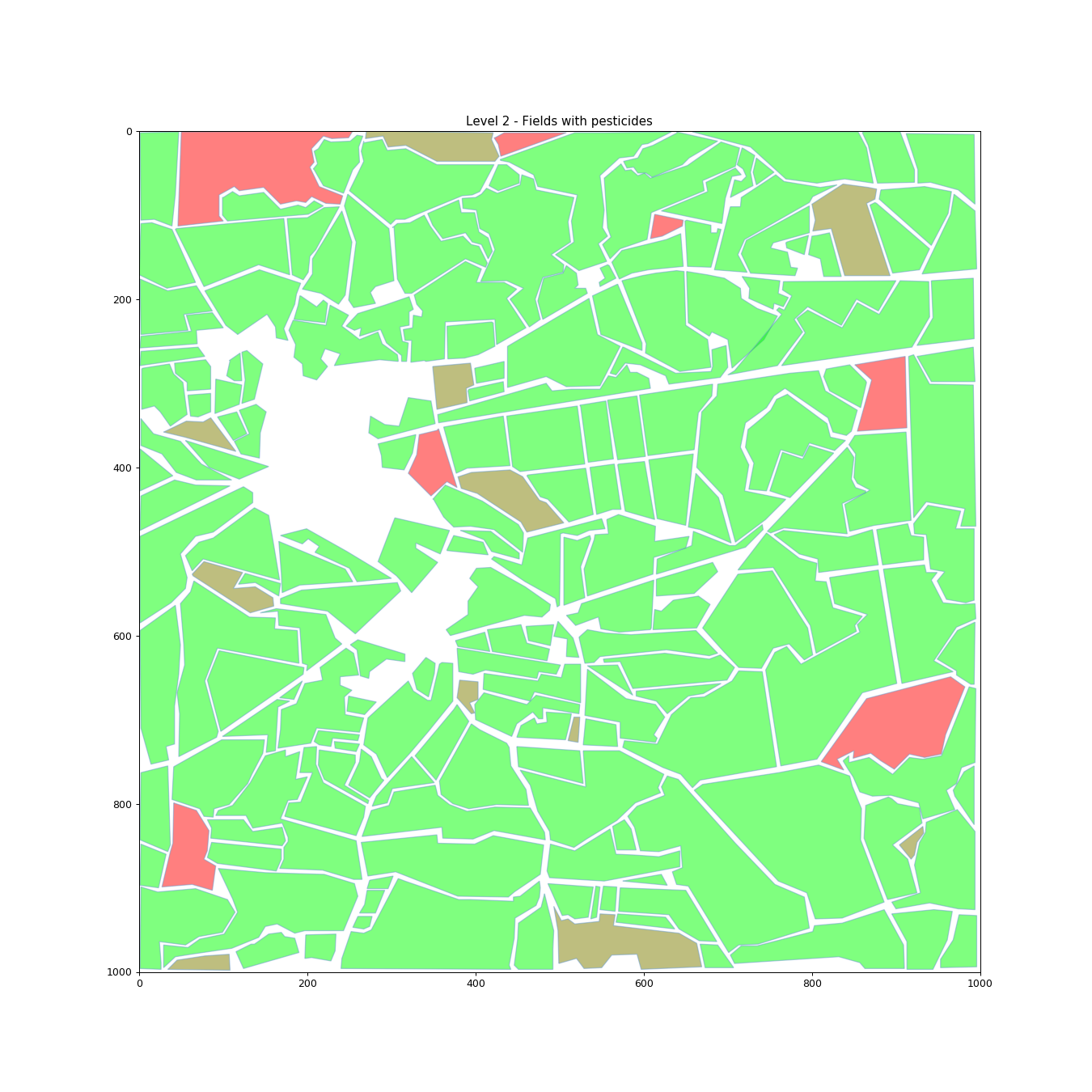

Supplement: Supplemental Information 1 — Python scripts developed to describe and run the model [file peerj-09-12178-s001.zip › Optimal Hives Amount in Point - Model/ClassLevelImages/Level 2 - Fields with pesticides.png]

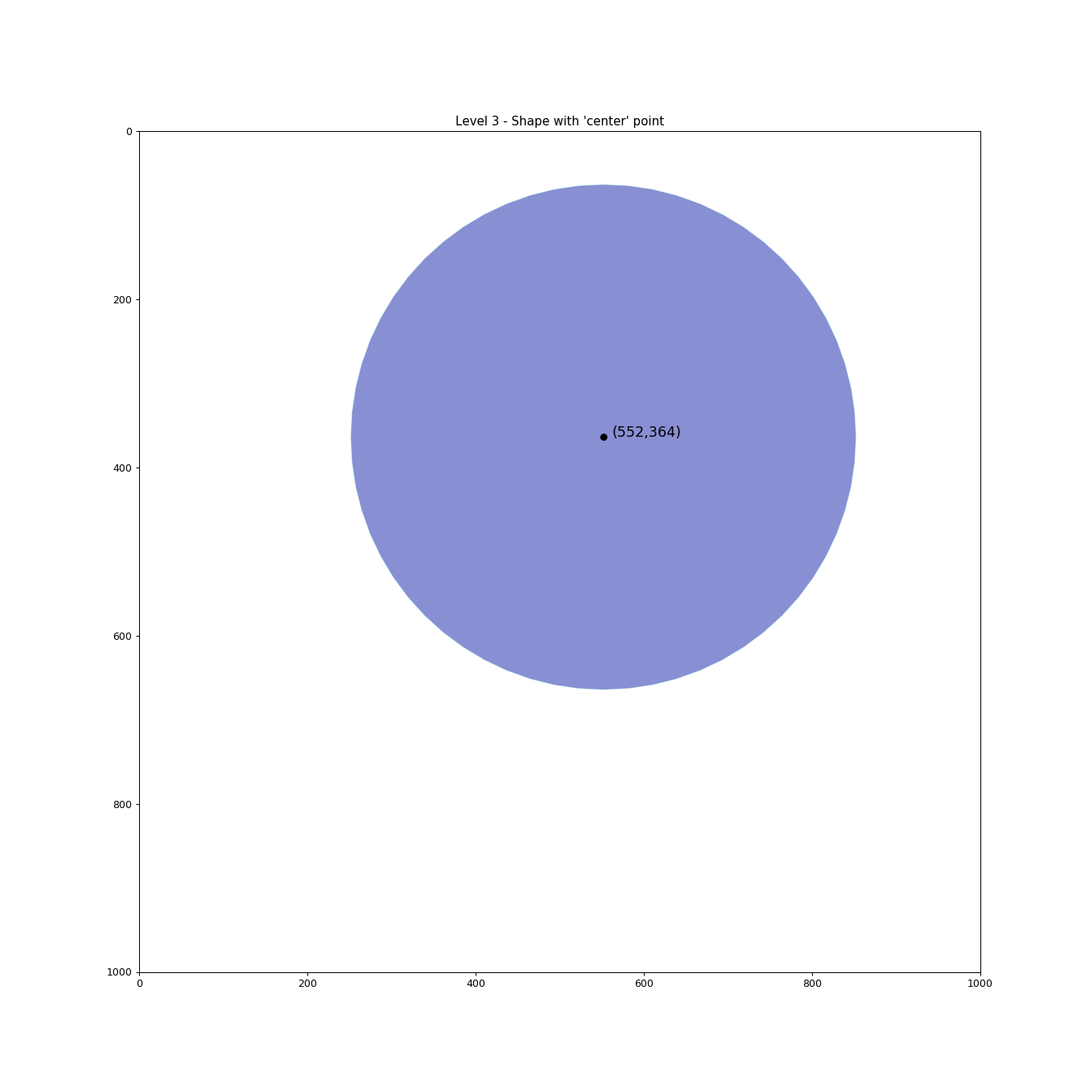

Supplement: Supplemental Information 1 — Python scripts developed to describe and run the model [file peerj-09-12178-s001.zip › Optimal Hives Amount in Point - Model/ClassLevelImages/Level 3 - Shape with 'center' point.png]

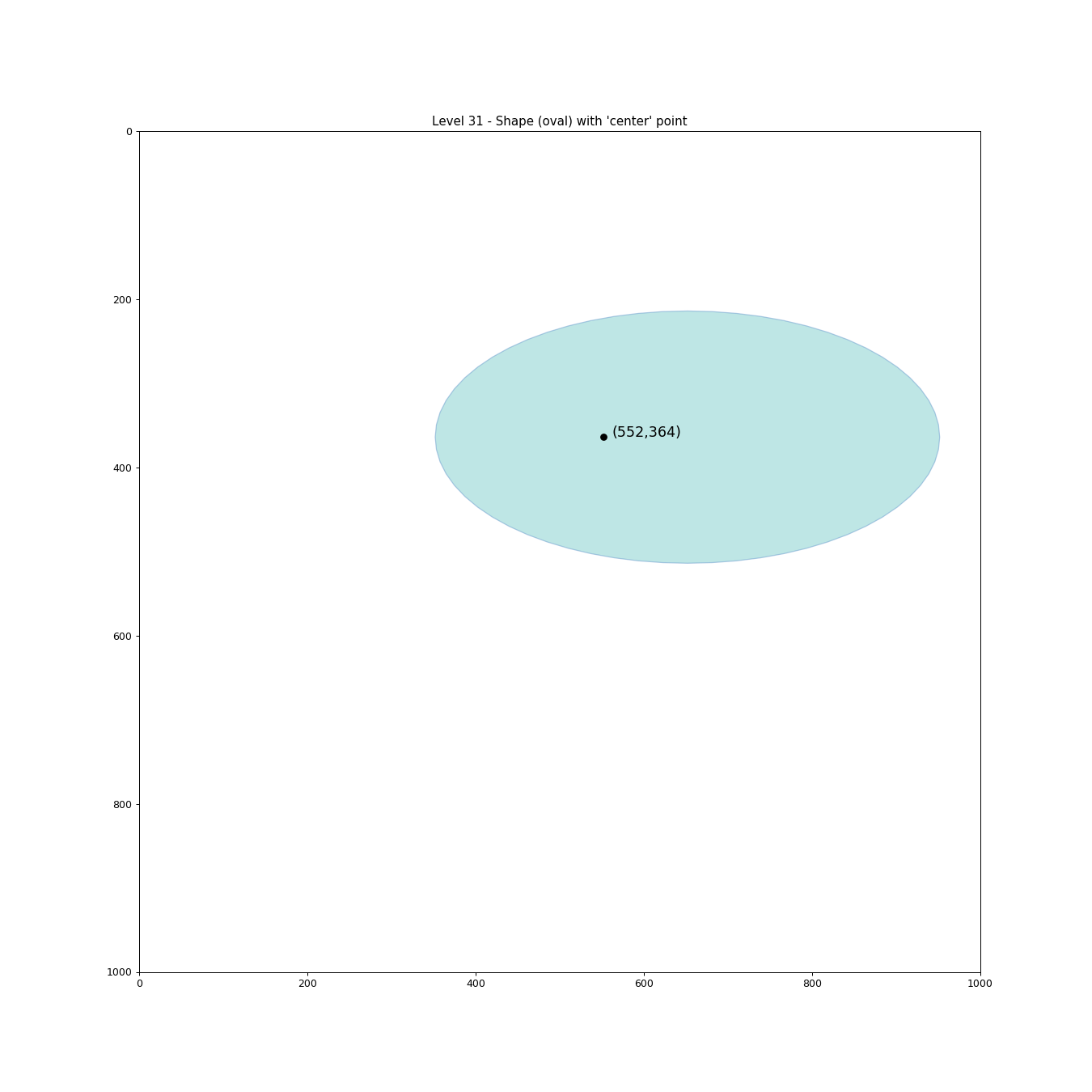

Supplement: Supplemental Information 1 — Python scripts developed to describe and run the model [file peerj-09-12178-s001.zip › Optimal Hives Amount in Point - Model/ClassLevelImages/Level 31 - Shape (oval) with 'center' point.png]

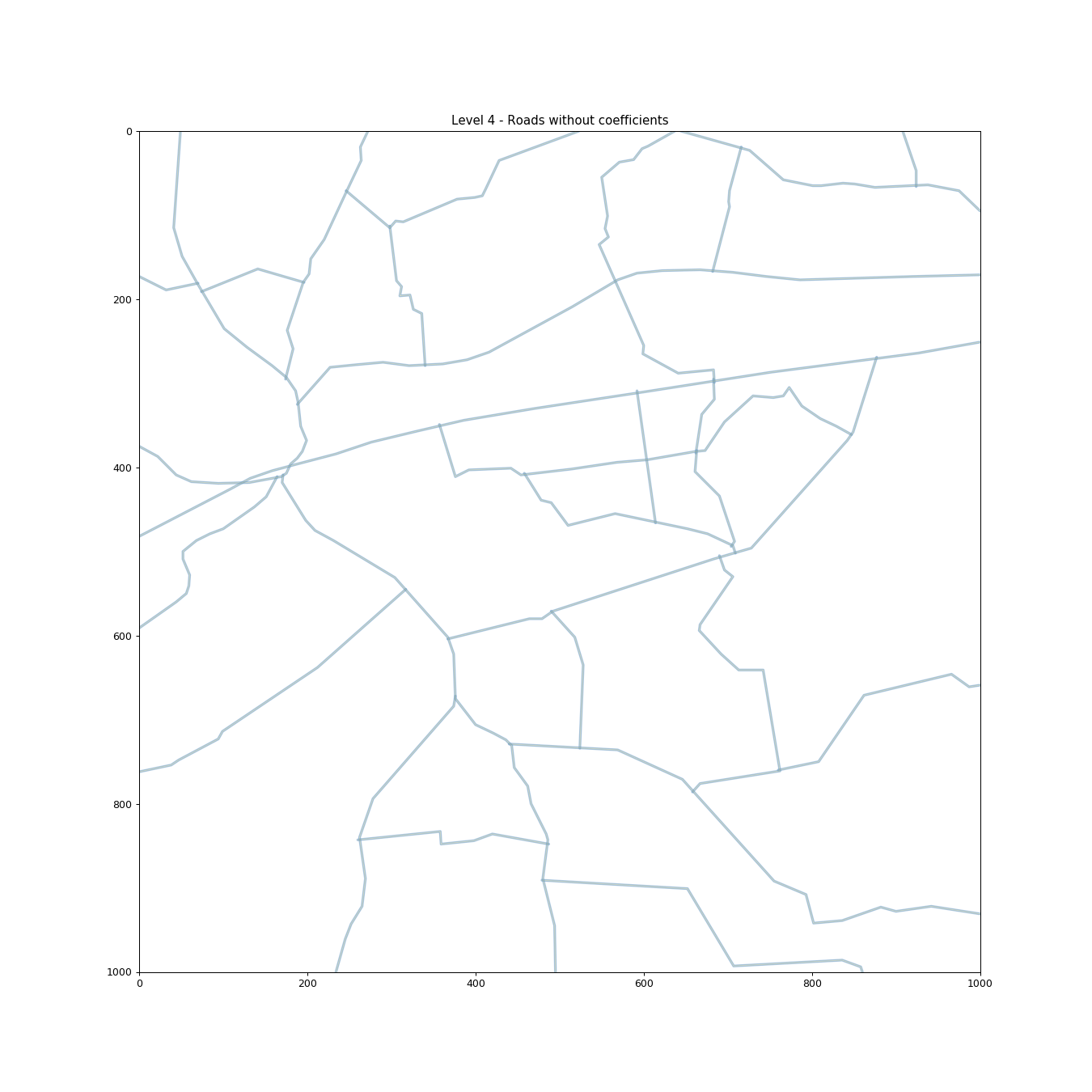

Supplement: Supplemental Information 1 — Python scripts developed to describe and run the model [file peerj-09-12178-s001.zip › Optimal Hives Amount in Point - Model/ClassLevelImages/Level 4 - Roads without coefficients.png]

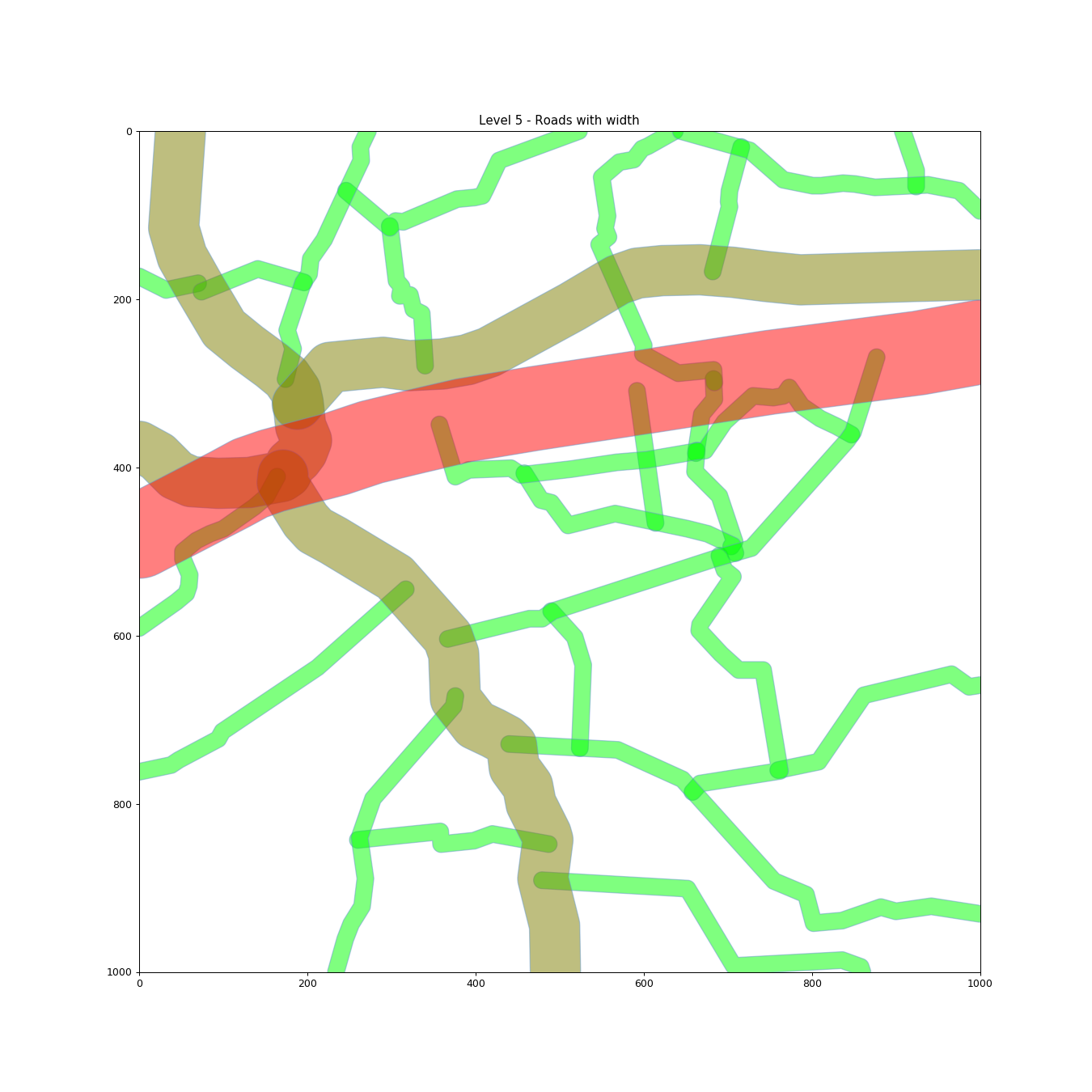

Supplement: Supplemental Information 1 — Python scripts developed to describe and run the model [file peerj-09-12178-s001.zip › Optimal Hives Amount in Point - Model/ClassLevelImages/Level 5 - Roads with width.png]

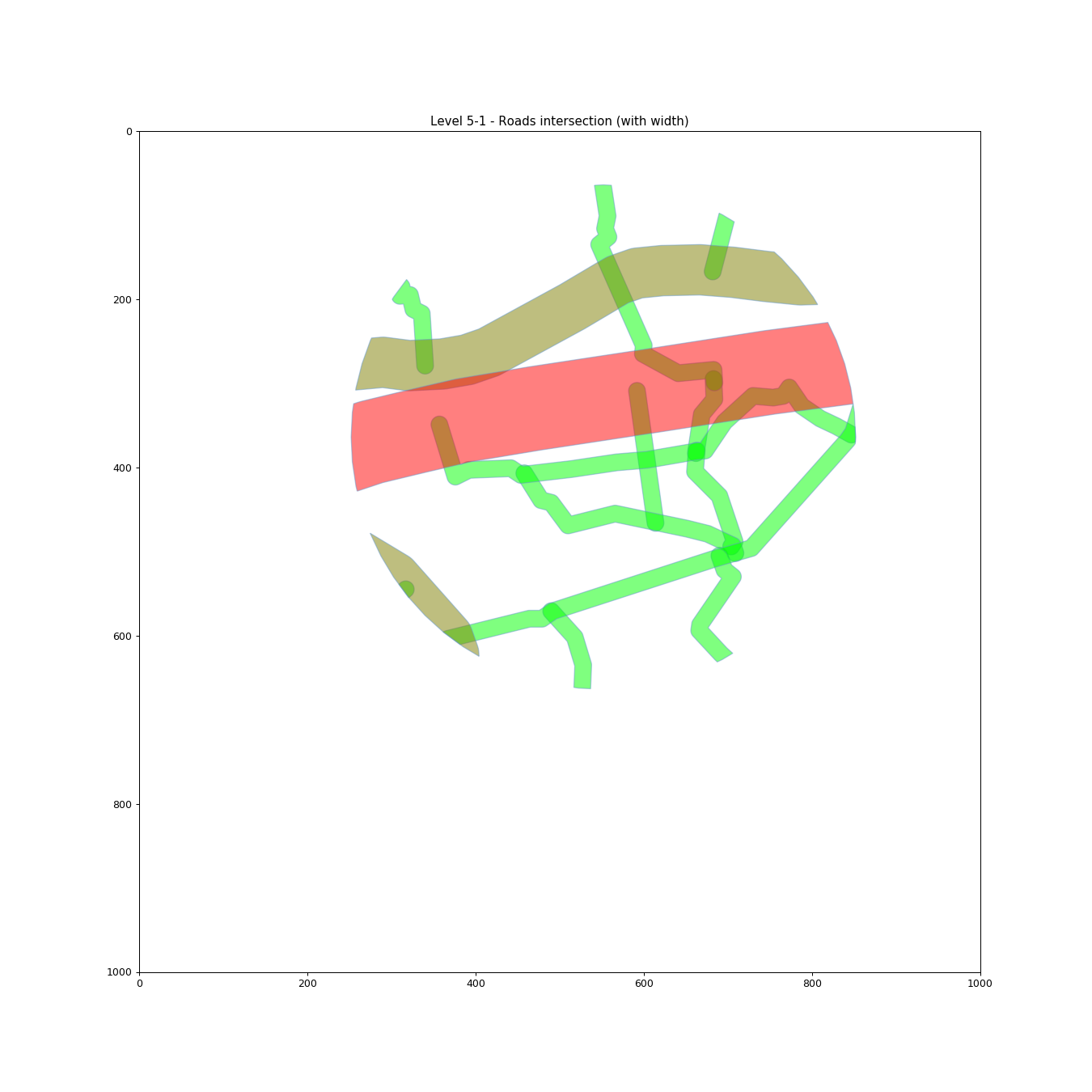

Supplement: Supplemental Information 1 — Python scripts developed to describe and run the model [file peerj-09-12178-s001.zip › Optimal Hives Amount in Point - Model/ClassLevelImages/Level 5-1 - Roads intersection (with width).png]

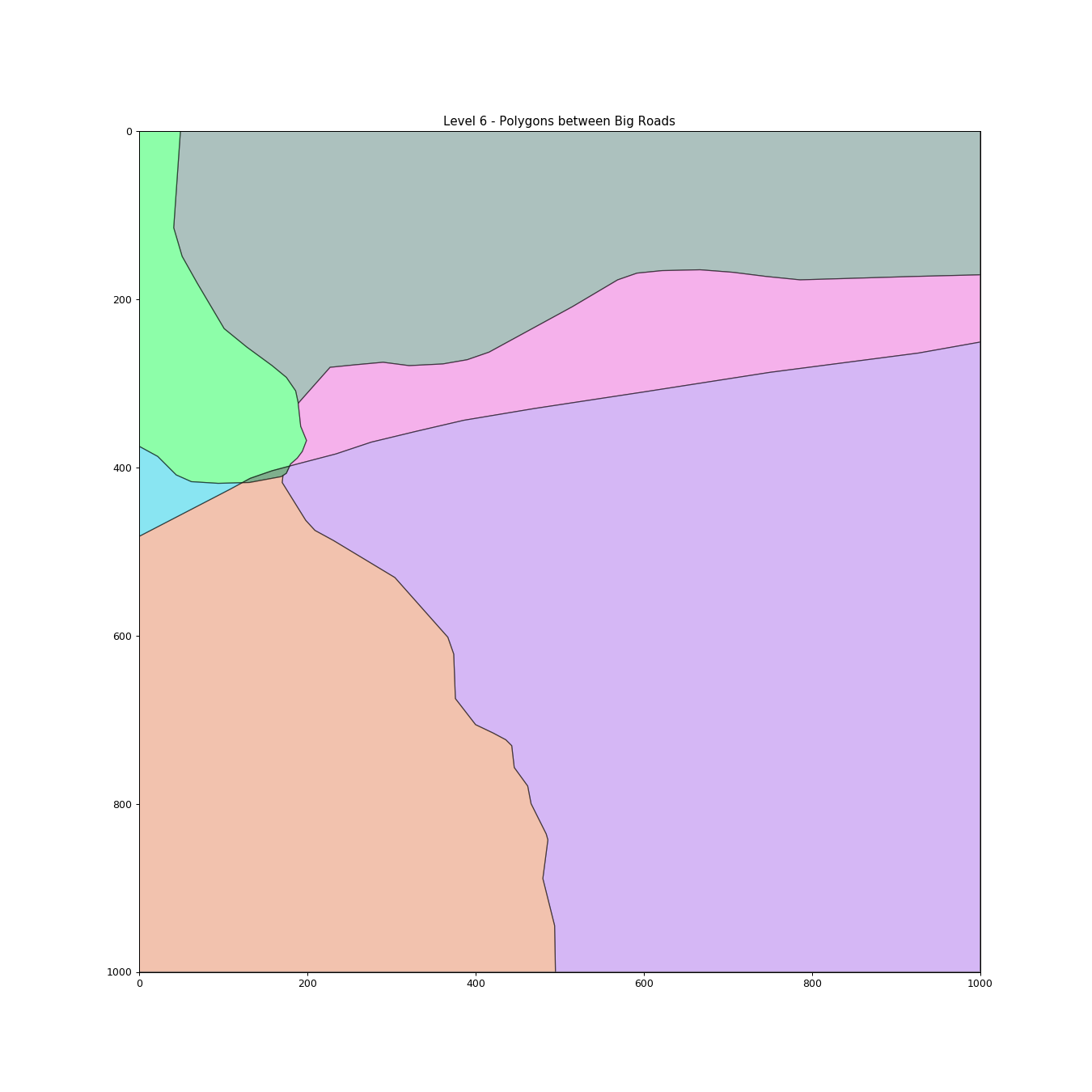

Supplement: Supplemental Information 1 — Python scripts developed to describe and run the model [file peerj-09-12178-s001.zip › Optimal Hives Amount in Point - Model/ClassLevelImages/Level 6 - Polygons between Big Roads.png]

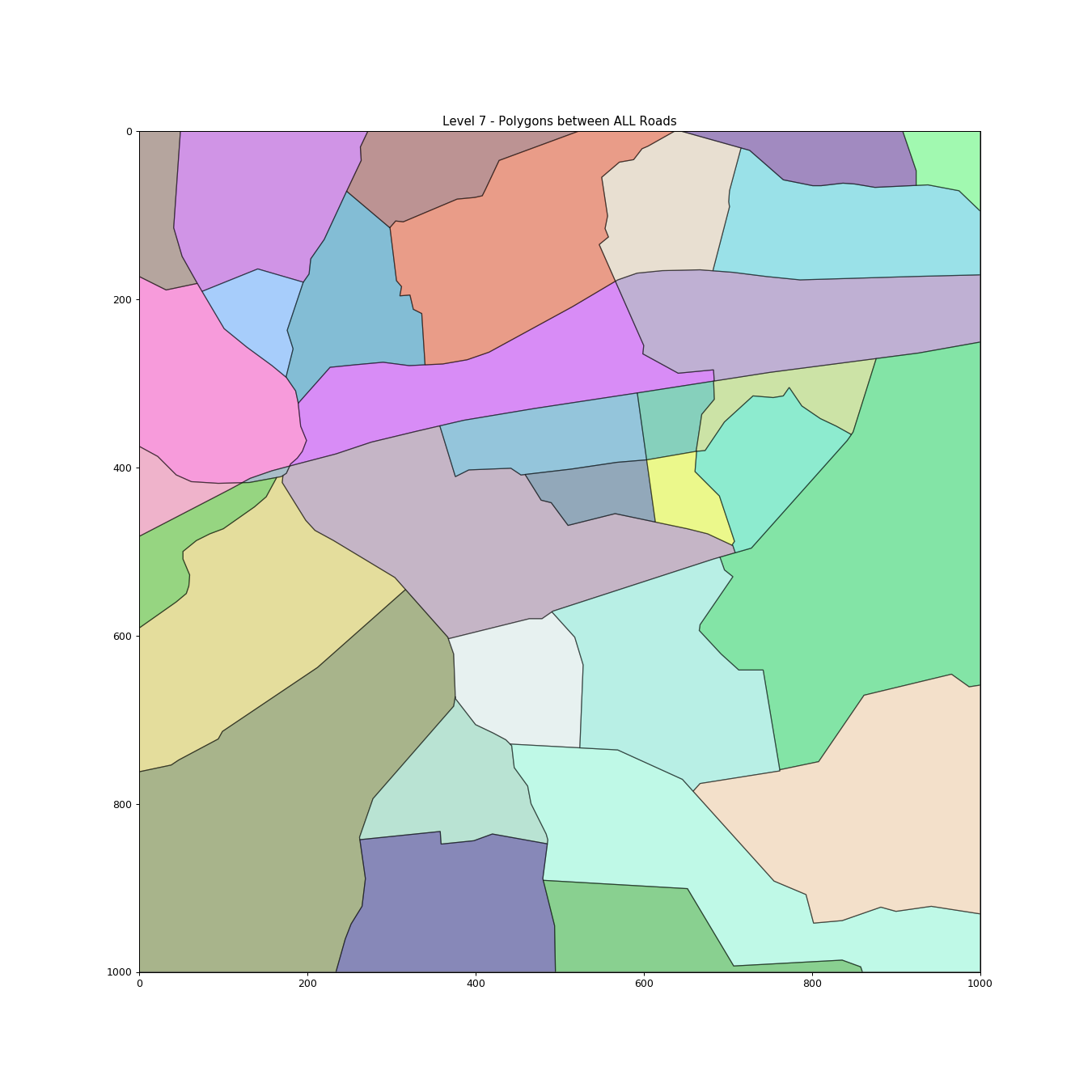

Supplement: Supplemental Information 1 — Python scripts developed to describe and run the model [file peerj-09-12178-s001.zip › Optimal Hives Amount in Point - Model/ClassLevelImages/Level 7 - Polygons between ALL Roads.png]

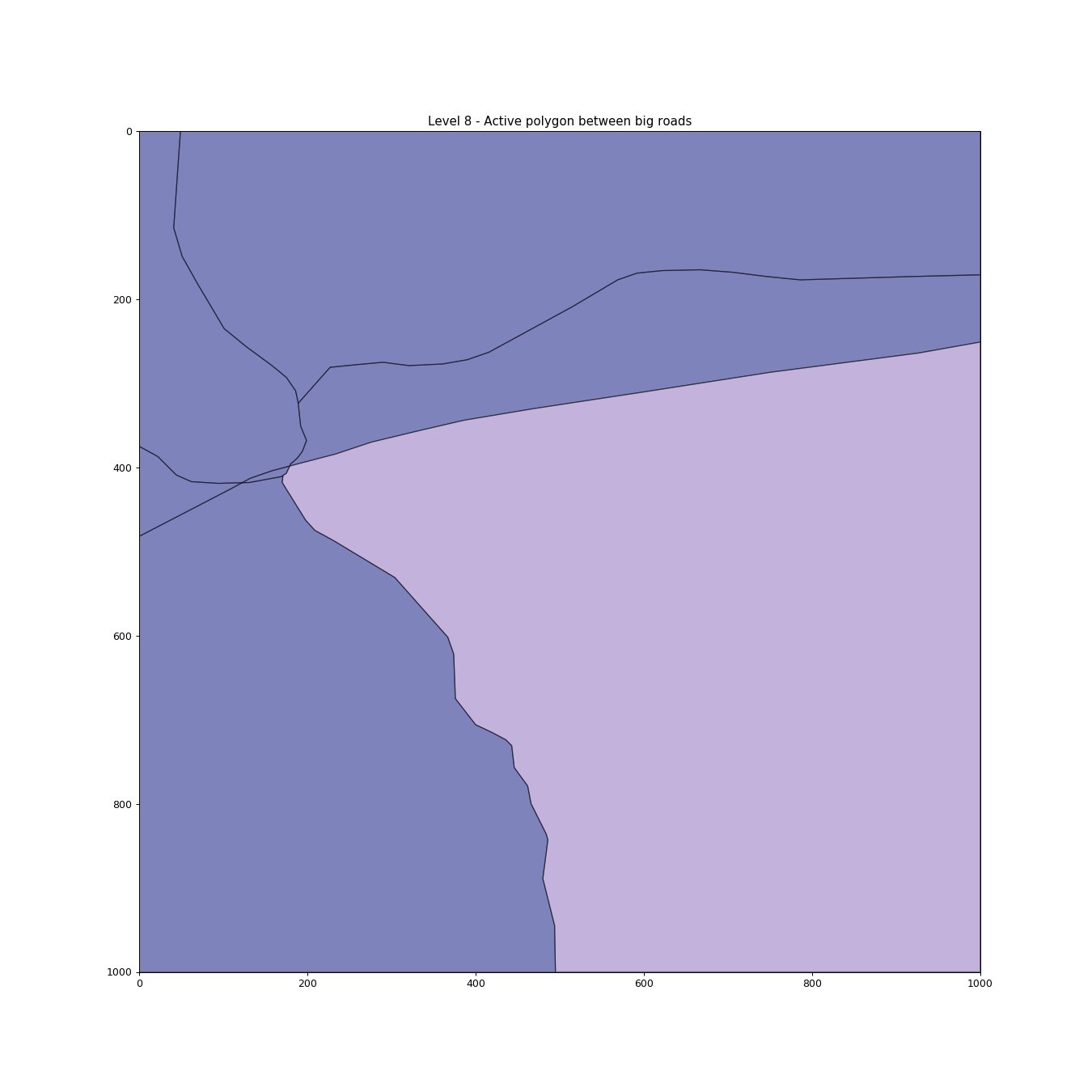

Supplement: Supplemental Information 1 — Python scripts developed to describe and run the model [file peerj-09-12178-s001.zip › Optimal Hives Amount in Point - Model/ClassLevelImages/Level 8 - Active polygon between big roads.png]

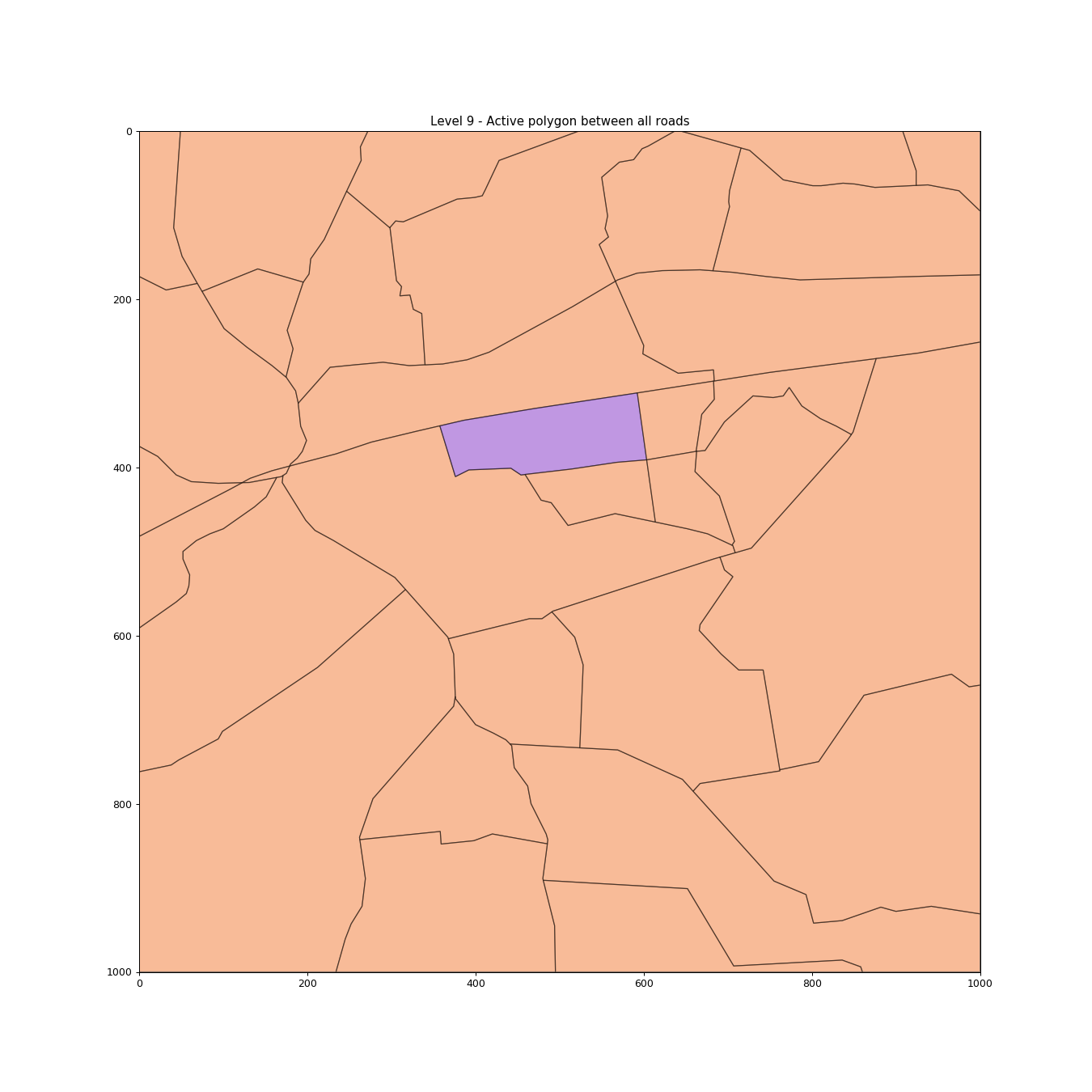

Supplement: Supplemental Information 1 — Python scripts developed to describe and run the model [file peerj-09-12178-s001.zip › Optimal Hives Amount in Point - Model/ClassLevelImages/Level 9 - Active polygon between all roads.png]

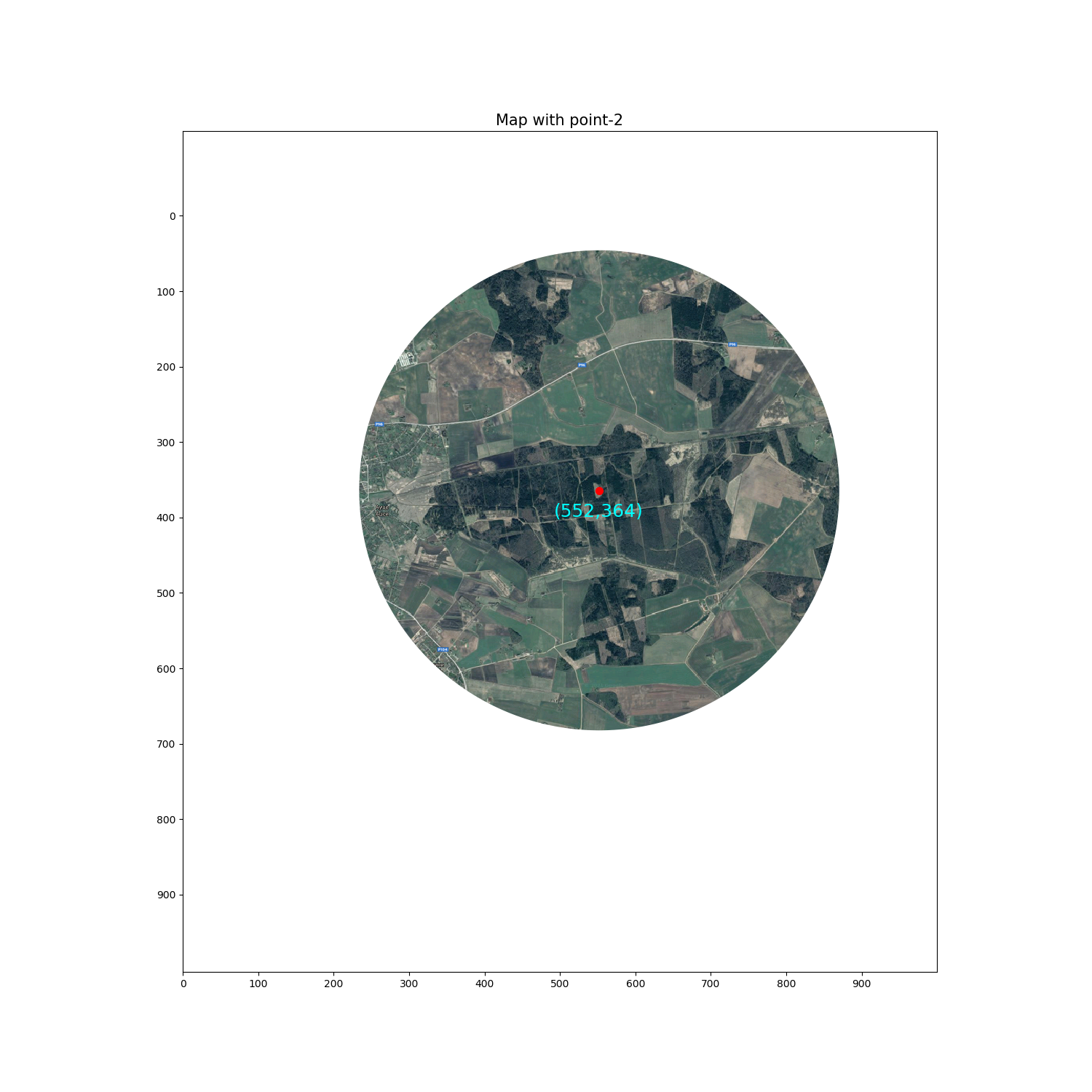

Supplement: Supplemental Information 1 — Python scripts developed to describe and run the model [file peerj-09-12178-s001.zip › Optimal Hives Amount in Point - Model/ClassLevelImages/Map with point-2.png]

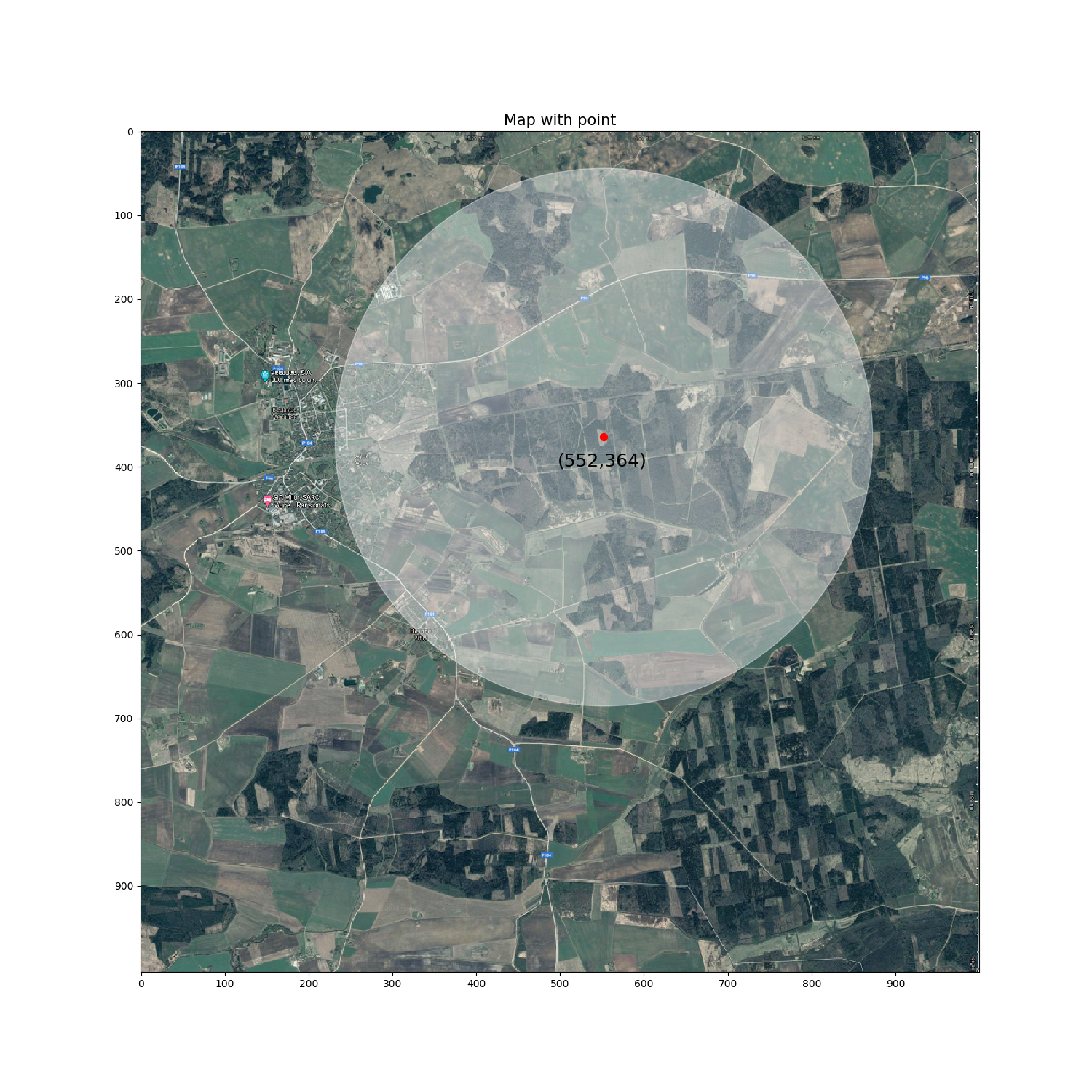

Supplement: Supplemental Information 1 — Python scripts developed to describe and run the model [file peerj-09-12178-s001.zip › Optimal Hives Amount in Point - Model/ClassLevelImages/Map with point.png]

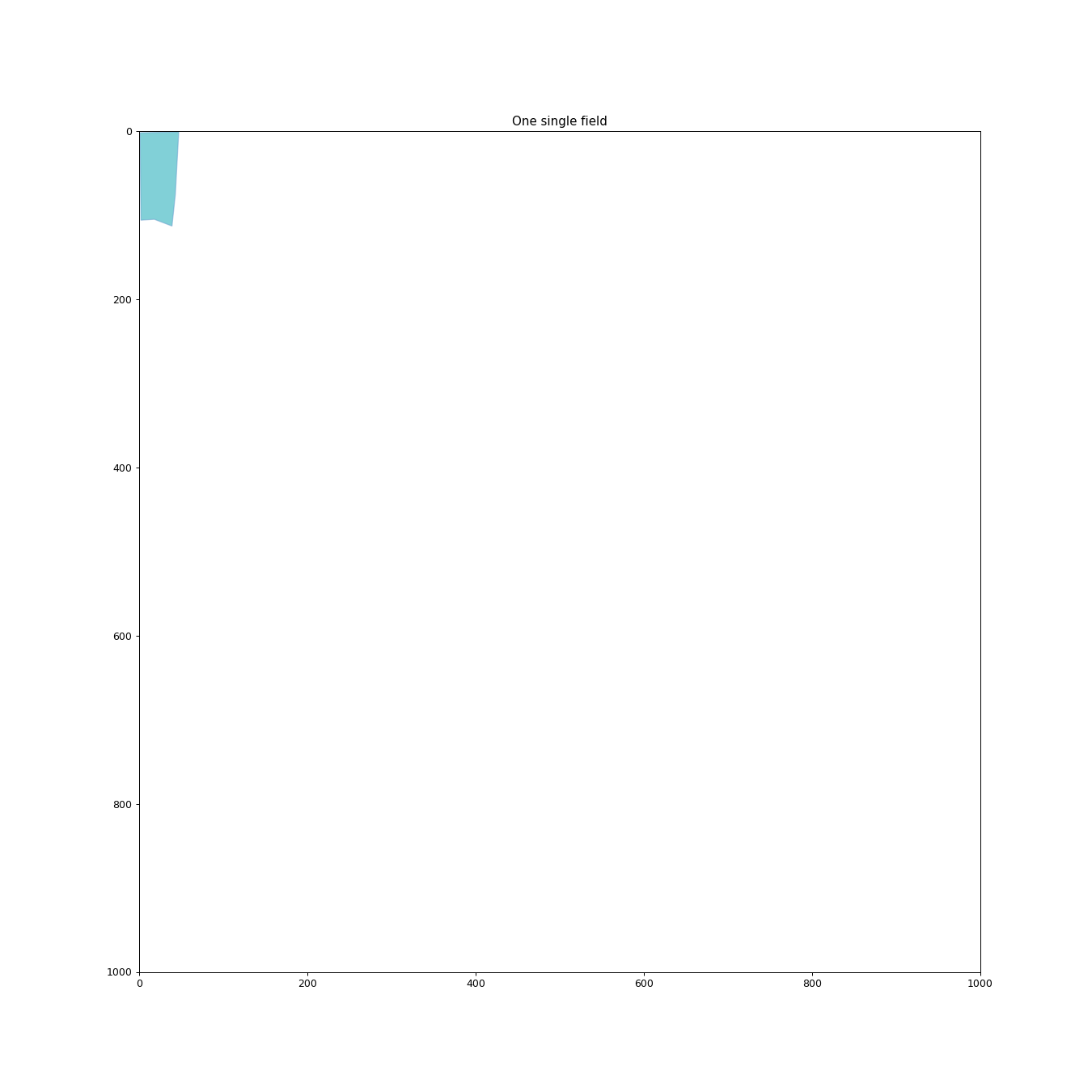

Supplement: Supplemental Information 1 — Python scripts developed to describe and run the model [file peerj-09-12178-s001.zip › Optimal Hives Amount in Point - Model/ClassLevelImages/One single field.png]

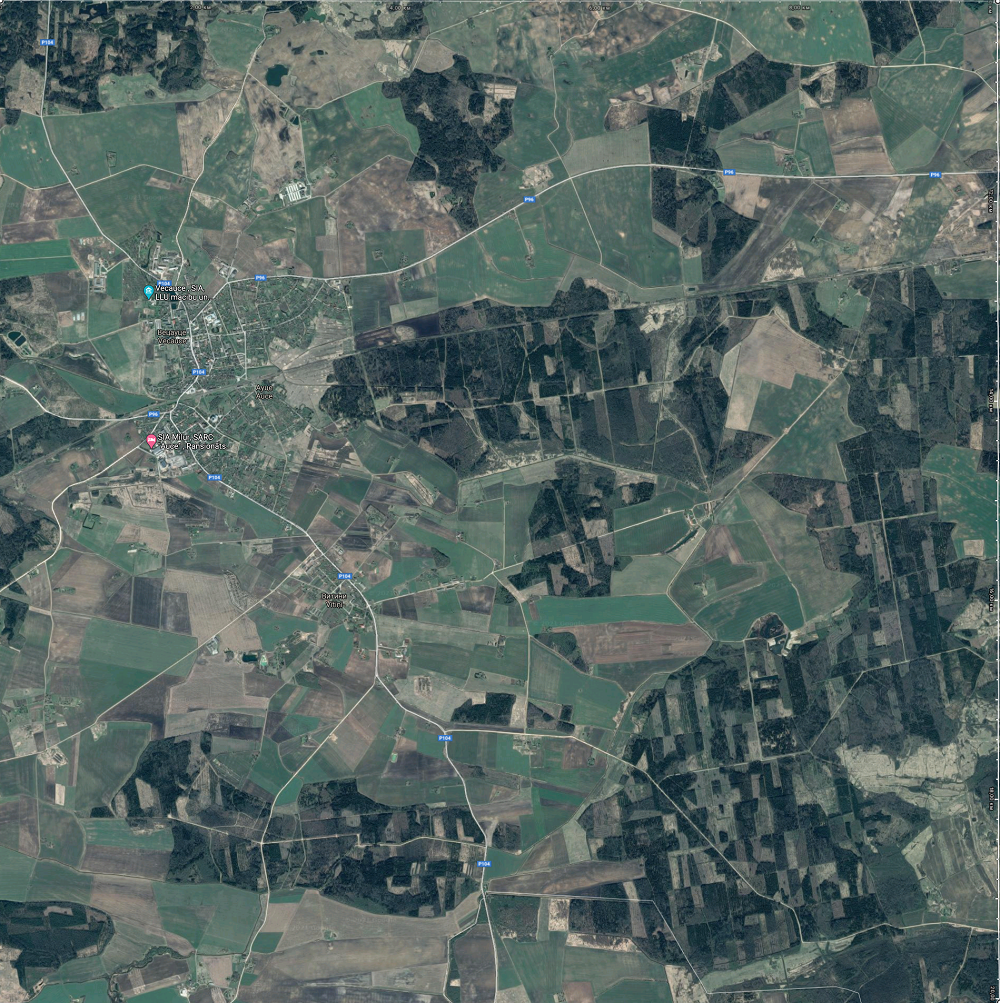

Supplement: Supplemental Information 1 — Python scripts developed to describe and run the model [file peerj-09-12178-s001.zip › Optimal Hives Amount in Point - Model/map_1000_1000.png]
